# N6-methyladenosine modification of MEF2A weakens cetuximab sensitivity in colorectal cancer via PD-L1/SOX12 axis

**DOI:** 10.1038/s41420-025-02577-8

**Published:** 2025-07-01

**Authors:** Cao Gao, Jiajia He, Jiemin Zhao, Xuefeng Ni, Yanjie Xu

**Affiliations:** 1 Department of Anesthesiology, The Third Affiliated Hospital of Soochow University, Changzhou, Jiangsu Province PR China; 2 Department of Oncology, The Third Affiliated Hospital of Soochow University, Changzhou, Jiangsu Province PR China

**Keywords:** Chemical biology, Diseases

## Abstract

Colorectal cancer (CRC) treatment is still a challenge due to chemoresistance. We explored MEF2A function and underlying mechanism on cetuximab sensitivity in CRC. In this study, cancer tissues and adjacent non-cancerous samples were harvested from CRC patients. Cell viability, proliferation and apoptosis in CRC cells were tested by CCK-8, EdU, colony formation, and flow cytometry assays. The binding of MEF2A on the PD-L1 promoter was validated using luciferase reporter assay, CHIP, and EMSA, while the relationship of PD-L1 and SOX12 mRNA, as well as RBM15/IGF2BP1 and MEF2A mRNA, was verified by RIP, RNA pull-down, or FISH combined with immunofluorescence. m6A modification level of MEF2A mRNA was assayed by MeRIP. The expressions of key genes and proteins, including MEF2A, PD-L1, SOX12, RBM15, IGF2BP1, apoptosis- and cell cycle-related proteins, were determined with RT-qPCR, western blot, or immunohistochemistry. In vivo function of MEF2A was validated by establishing a xenograft nude mice model. The results showed that MEF2A was increased in CRC cells and tissues, while it was higher in cetuximab-resistant CRC tissues. Silencing MEF2A improved the sensitivity of cetuximab in CRC cells and xenograft mice. MEF2A binds to PD-L1 promoter to transcriptionally upregulate PD-L1 expression. Increased cetuximab sensitivity was observed in PD-L1 knockout (KO) CRC cells. PD-L1 overexpression reversed the enhanced cetuximab sensitivity induced by MEF2A knockdown. PD-L1 binds to SOX12 mRNA to stabilize its expression. PD-L1 knockdown augmented cetuximab sensitivity, which was overturned by SOX12 overexpression. The m6A modification mediated by RBM15/IGF2BP1 upregulated MEF2A expression in cetuximab-resistant CRC tissues. In conclusion, m6A-modified MEF2A alleviated cetuximab sensitivity in CRC via PD-L1/SOX12 mRNA axis, indicating that MEF2A might function as a promising therapeutic target against cetuximab-resistant CRC.

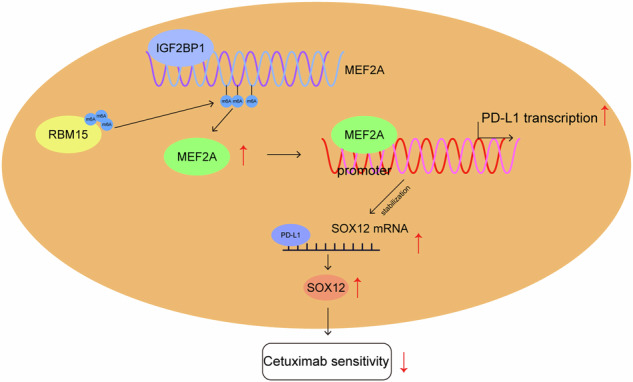

## Introduction

Colorectal cancer (CRC) is one of the major causes of cancer-associated morbidity and mortality in the world [1]. First-line managements for CRC, such as radiotherapy and chemotherapy, always produce dose-limiting toxicity in patients and cause resistance to cancer cells [2]. Cetuximab, an anti-epidermal growth factor receptor (EGFR) monoclonal antibody, is efficient for treating metastatic CRC [3]. However, resistance to chemotherapy impairs its clinical application and efficacy [4]. Thus, it is of great significance for exploring the mechanism of resistance to cetuximab and developing novel strategies for strengthening drug sensitivity in CRC.

Myocyte enhancer factor (MEF) 2A is a transcription factor belonging to MEF2 family [5]. MEF2A transcriptionally upregulates the expression levels of catenin beta 1 (CTNNB1) and zinc finger E-box binding homeobox 2 (ZEB2) in CRC to promote tumor progression [6]. Bioinformatics analysis (Kaplan-Meier Plotter) shows that higher MEF2A level is associated with the lower 3- and 5-year survivals of CRC patients. Also, MEF2A can positively regulate neurogenic locus notch homolog protein 3 (NOTCH3), which reduces docetaxel chemosensitivity in prostate cancer [7]. However, whether MEF2A regulates cetuximab sensitivity in CRC has not been clarified.

N6-methyladenosine (m6A) methylation is the most abundant mRNA modification in eukaryotes, which participates in the modulation of RNA transcription, translation, and degradation [8]. Studies have reported the underlying role of m6A modification on drug resistance in cancers [9]. Bioinformatics (RMBase) has predicted that MEF2A is modified by m6A, and ENCORI database also has shown both m6A “writer” RNA binding motif protein 15 (RBM15) and “reader” insulin-like growth factor 2 mRNA-binding protein 1 (IGF2BP1) can recognize MEF2A mRNA. Currently, the regulation of RBM15/IGF2BP1 on MEF2A m6A modification has not been verified. Furthermore, knockdown of RBM15 inhibits proliferation and invasion of CRC cells [10, 11] and alleviates paclitaxel resistance in ovarian cancer [12]. IGF2BP1 restrains tumor cytotoxicity mediated by CD8^+^ T cells in colon cancer [13]. Besides, IGF2BP1 overexpression promotes 5-fluorouracil and etoposide resistance in CRC cells [14]. However, the roles of RBM15 and IGF2BP1 in cetuximab sensitivity of CRC are still unknown.

Previously, we have demonstrated that programmed death ligand 1 (PD-L1), as a widely reported key factor regulating immune escape, is highly expressed in cancer tissues from cetuximab-resistant CRC patients and can mediate cetuximab resistance [15]. The relationship of MEF2A and PD-L1 has not been clarified so far. JASPAR database displays there are binding sequences between MEF2A and the promoter region of PD-L1. Furthermore, PD-L1 functions as an RNA-binding protein that stabilizes target mRNAs, and RIP-seq and RNA-seq data present that PD-L1 can bind to the mRNA of sex-determining region Y-box (SOX) 12 [16, 17]. SOX12, as a cancer stem cell marker, is highly expressed in CRC; besides, overexpression of SOX12 facilitates the proliferation and migration of CRC cells [18, 19]. SOX12 can promote cisplatin resistance in hepatocellular carcinoma [20]. Also, cetuximab resistance is promoted in CRC via upregulating SOX2 expression [21]. However, whether MEF2A/PD-L1 axis regulates cetuximab sensitivity via stabilizing SOX12 mRNA needs to be validated.

The current research explored the regulatory function of MEF2A on cetuximab sensitivity in CRC, as well as further explored the underlying mechanism. We demonstrated that RBM15/IGF2BP1-mediated m6A modification of MEF2A upregulated its expression to promote PD-L1 transcription, which stabilized SOX12 mRNA and then weakened cetuximab sensitivity in CRC. The present study provided a potential novel target to overcome cetuximab resistance in CRC.

## Results

### MEF2A was increased in CRC tissues, cetuximab-resistant CRC tissues, and CRC cell lines

**Fig. 1:**
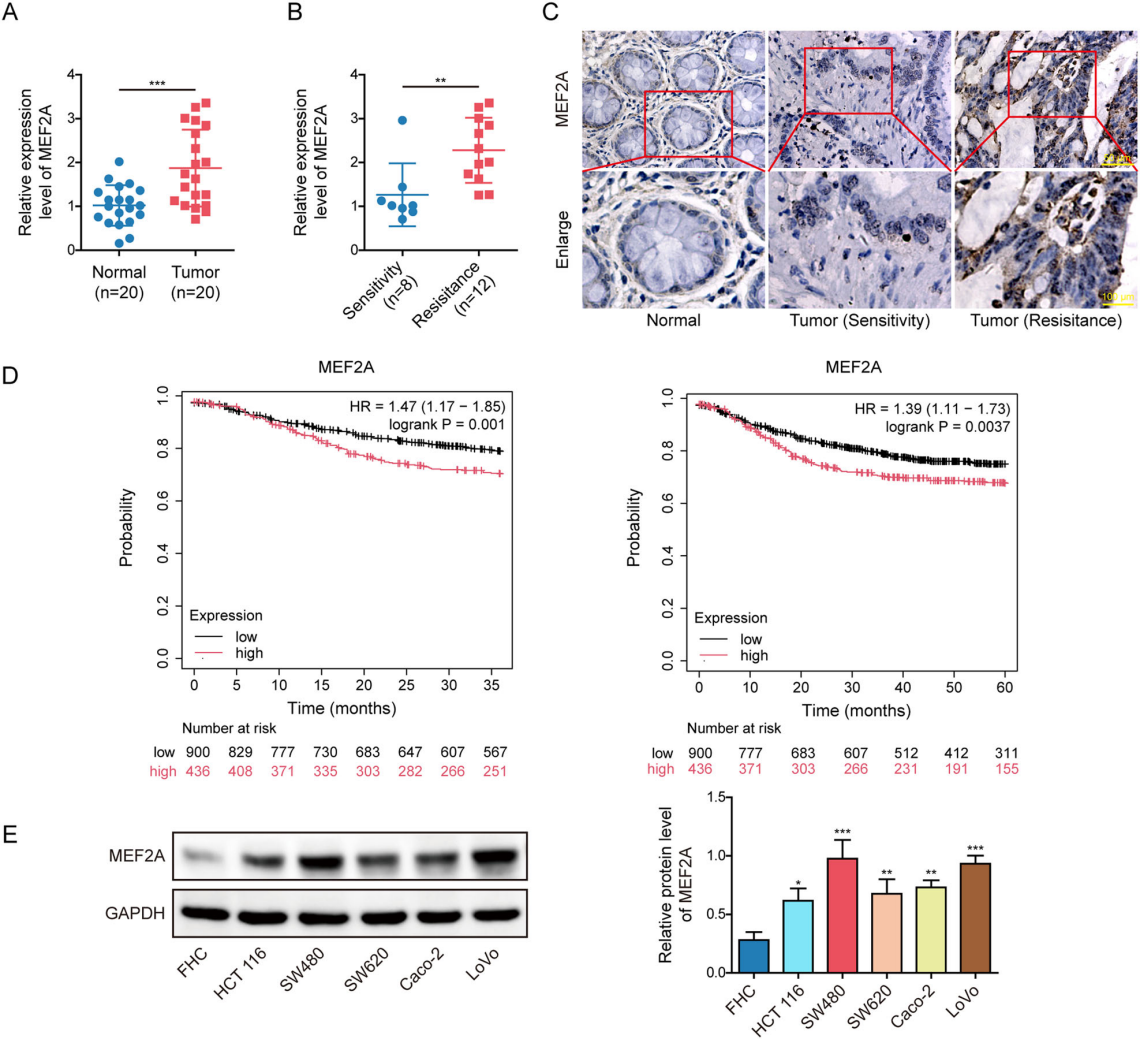
MEF2A was increased in CRC tissues, cetuximab-resistant CRC tissues, as well as CRC cell lines. CRC patients (*n* = 20, including 8 cetuximab-sensitive and 12 cetuximab-resistant specimens) were recruited for the collection of cancerous tissues and adjacent non-cancerous tissues. RT-qPCR for measuring MEF2A mRNA levels in normal tissues and cancerous tissues (**A**), as well as in cetuximab-resistant and cetuximab-sensitive CRC tissues (B). MEF2A protein level in clinical samples was detected with immunohistochemistry. Scale bar: 50 μm and 10 μm (**C**). **D** The association between 3-year and 5-year survival rates of CRC patients and MEF2A level showed by bioinformatics analysis (Kaplan–Meier Plotter). **E** In normal colonic epithelial cell line (FHC) and CRC cell lines (HCT 116, SW480, SW620, Caco-2, and LoVo), MEF2A protein levels were determined with western blot. All bar chart analysis of western blot is a repeated experiment three times. *p < 0.05, **p < 0.01, and ***p < 0.001.

Initially, RT-qPCR results presented that in comparison with adjacent non-cancerous specimens, MEF2A mRNA levels were increased in CRC tissues (Fig. 1A). Moreover, as shown in Fig. 1B, MEF2A mRNA levels in cetuximab-resistant CRC tissues were higher than those of sensitive CRC samples. Consistently, immunohistochemical results also presented that MEF2A protein was increased in cetuximab-sensitive CRC specimens in comparison to adjacent normal tissues, while it was further upregulated in resistant CRC tissues (Fig. 1C). Bioinformatics analysis (Kaplan-Meier Plotter) showed CRC patients with higher MEF2A level had lower 3- and 5-year survival rates (Fig. 1D). Compared to that in normal colonic epithelial cell line (FHC), the protein levels of MEF2A were found to be upregulated in CRC cell lines including HCT 116, SW480, SW620, Caco-2, and LoVo (Fig. 1E). These results verified high MEF2A expression in CRC tissues, cetuximab-resistant CRC tissues, as well as CRC cell lines.

### MEF2A knockdown enhanced cetuximab chemosensitivity in CRC cells

**Fig. 2:**
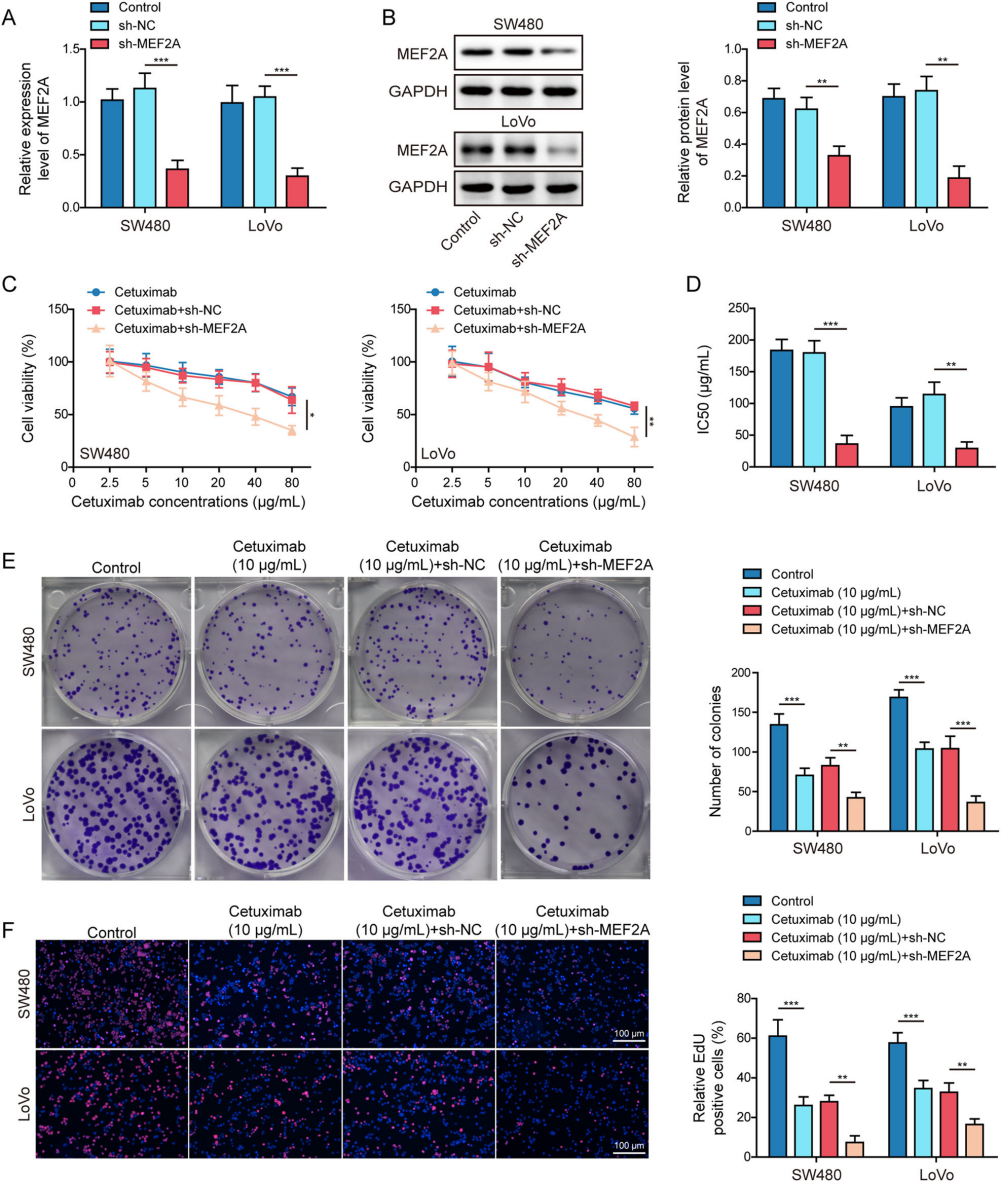
MEF2A knockdown enhanced cetuximab sensitivity in CRC cells. **A**, **B** sh-MEF2A was transfected into CRC cells (SW480 and LoVo) for 48 h. MEF2A mRNA and protein expressions were determined using RT-qPCR (**A**) and western blot (**B**). CRC cells were transfected with sh-MEF2A, before cetuximab exposure. CCK-8 assay was used for measuring cell viability (**C**), and IC50 values were calculated (**D**). Clonal formation assay (**E**) and EdU staining (**F**) for detecting cell proliferation. Scale bar: 100 μm. All bar chart analysis of western blot is a repeated experiment three times. *p < 0.05, **p < 0.01, and ***p < 0.001.

We then identified the function of MEF2A on the response of CRC cells to cetuximab. Two CRC cell lines (SW480 and LoVo) with relatively higher MEF2A expression were selected for following experiments. Decreased mRNA (Fig. 2A) and protein (Fig. 2B) expression levels of MEF2A were observed in sh-MEF2A-transfected CRC cells, indicating successful knockdown of MEF2A. Subsequently, CRC cells transfected with sh-MEF2A were subjected to cetuximab treatment, and their sensitivities to cetuximab were determined. Both cell viability (Fig. 2C) and IC50 value (Fig. 2D) for cetuximab were decreased following MEF2A silencing, compared to those of cetuximab+sh-NC group. The amount of clones (Fig. 2E) as well as the proportion of EdU-positive cells (Fig. 2F) were reduced following cetuximab treatment, which were further declined by MEF2A depletion. Cetuximab elevated the apoptosis rate in CRC cells, moreover, knockdown of MEF2A enhanced this effect (Fig. S1A). CRC cells treated with cetuximab showed an increase in apoptosis-related protein cleaved caspase-3 and a decrease in uncleaved PARP, as well as reductions in cell cycle-associated proteins including CDK2, cyclin E1, and cyclin D1 (Fig. S1B). Furthermore, the outcomes induced by cetuximab were strengthened following MEF2A silencing (Fig. S1B). Collectively, these data revealed MEF2A silencing augmented the sensitivity of CRC cells to cetuximab.

### MEF2A bound to the PD-L1 promoter to transcriptionally upregulate PD-L1 expression

**Fig. 3:**
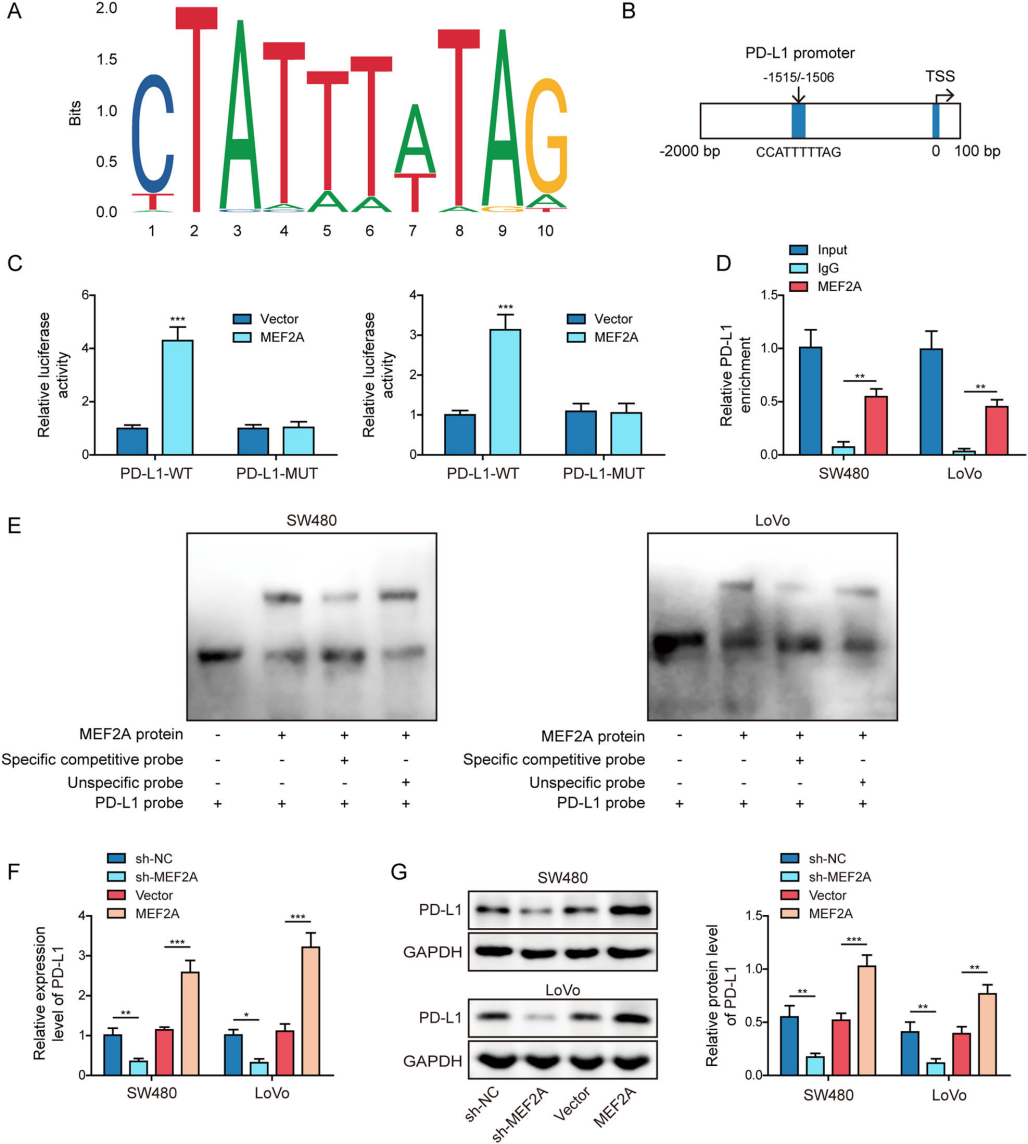
MEF2A bound to PD-L1 promoter to transcriptionally upregulate PD-L1 expression. The binding motif for MEF2A (**A**) and MEF2A binding sequences at PD-L1 promoter region (**B**) were forecasted through JASPAR database. Luciferase reporter assay (**C**), CHIP (**D**), and EMSA (**E**) for analyzing the binding of MEF2A protein on PD-L1 promoter in CRC cells. **F**, **G** sh-MEF2A or MEF2A overexpression vector was transfected into CRC cells. RT-qPCR (b**F**) and western blot (**G**) for testing PD-L1 mRNA and protein expressions. All bar chart analysis of western blot is a repeated experiment three times. *p < 0.05, **p < 0.01, and ***p < 0.001.

The potential mechanism of MEF2A-induced cetuximab resistance was further examined. The binding motif for transcription factor MEF2A (Fig. 3A) and putative MEF2A binding sites at PD-L1 promoter region (Fig. 3B) were identified by using JASPAR database. Thereby, we then elucidated whether MEF2A transcriptionally modulated PD-L1 expression. The luciferase activity driven by the mutant PD-L1 promoter (PD-L1-MUT) was not affected by overexpressing MEF2A, but MEF2A overexpression boosted the luciferase activity driven by the wild-type PD-L1 promoter (PD-L1-WT) (Fig. 3C). CHIP assay revealed that the enrichment of the sequences at PD-L1 promoter region was observed in the MEF2A-precipitated DNA fragments (Fig. 3D). EMSA assay confirmed that probe of the PD-L1 promoter region could bind to MEF2A protein (Fig. 3E). These results validated the binding of MEF2A to PD-L1 promoter region. In addition, overexpression or knockdown of MEF2A, respectively, up-regulated or down-regulated the mRNA (Fig. 3F) and protein (Fig. 3G) levels of PD-L1 in comparison with corresponding control group. Taken together, these findings suggested that MEF2A bound to PD-L1 promoter region to transcriptionally upregulate PD-L1 expression in CRC cells.

### MEF2A silencing promoted the sensitivity of CRC cells to cetuximab via restraining PD-L1 transcription

To validate the role of PD-L1 in cetuximab sensitivity to CRC, PD-L1 knockout (KO) cell lines (SW480-KO and LoVo-KO) were generated from CRC cell lines (SW480 and LoVo). PD-L1 protein was significantly expressed in CRC cell lines, but not in PD-L1 KO cell lines (Fig. S2A), indicating effective knockout of PD-L1. In the presence of cetuximab, knocking out PD-L1 in CRC cell lines obviously decreased cell viability (Fig. S2B), inhibited proliferation (Fig. S2C), promoted apoptosis (Fig. S2D), and led to an increase of cleaved caspase-3 and a decrease of uncleaved PARP (Fig. S2E). The findings suggested enhanced cetuximab sensitivity in PD-L1 knockout CRC cells.

**Fig. 4:**
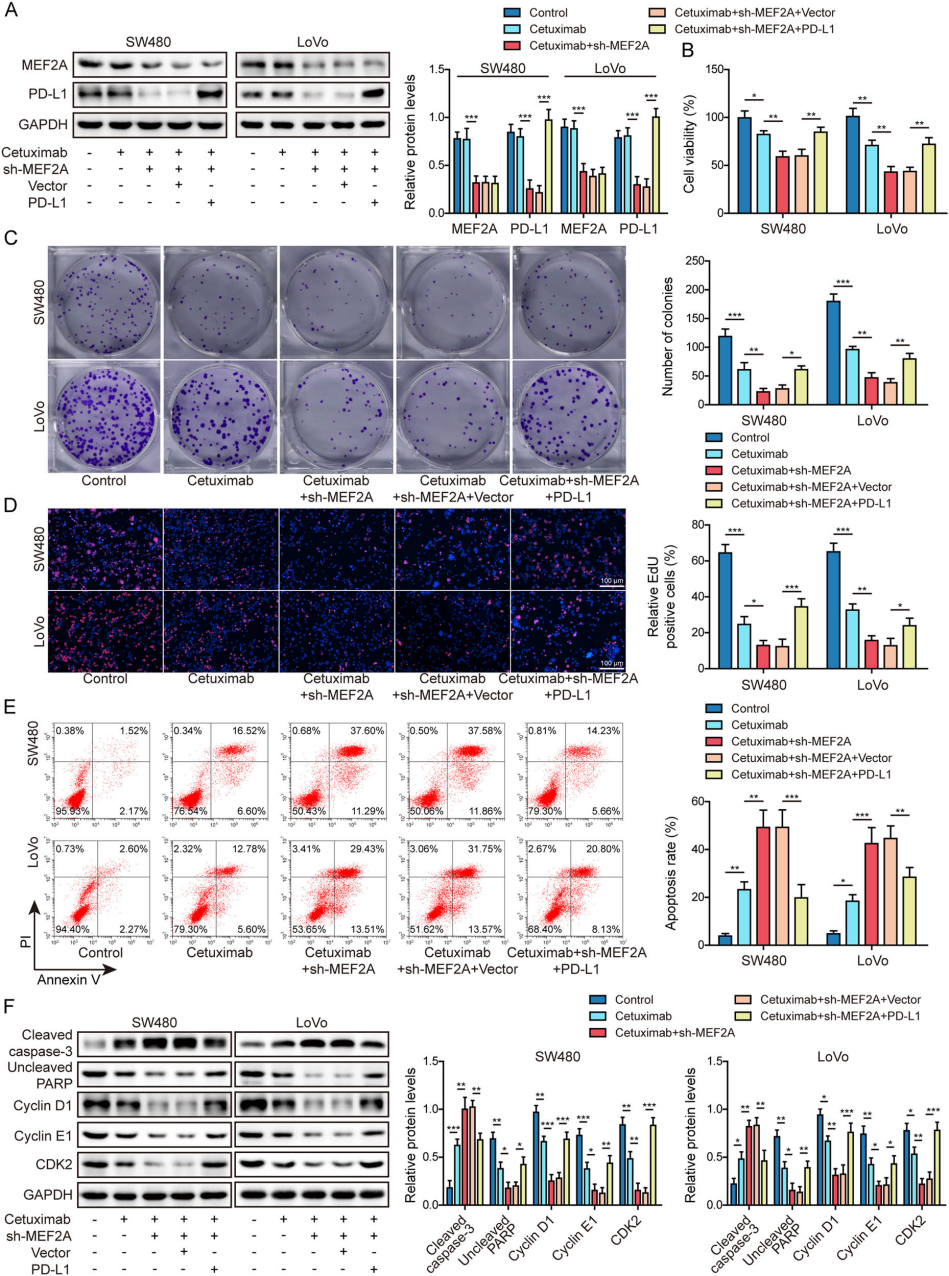
Knockdown of MEF2A boosted cetuximab sensitivity in CRC by inhibiting PD-L1 transcription. CRC cells transfected with sh-MEF2A or PD-L1 overexpression vector were exposed to cetuximab. **A** Western blot was performed for measuring MEF2A and PD-L1 protein expressions. **B** CCK-8 for measuring cell viability. Cell proliferation was detected with clonal formation assay (**C**) and EdU staining (**D**). Scale bar: 100 μm. **E** Flow cytometry for assessing apoptosis. **F** Western blot for measuring cleaved caspase-3, uncleaved PARP, cyclin D1, cyclin E1, and CDK2. All bar chart analysis of western blot is a repeated experiment three times. *p < 0.05, **p < 0.01, and ***p < 0.001.

To further determine whether PD-L1 was indispensable for MEF2A-mediated cetuximab sensitivity, sh-MEF2A and PD-L1 overexpression vector were co-transfected into CRC cells before cetuximab treatment. In comparison to control group, the expressions of MEF2A and PD-L1 were not significantly altered by cetuximab exposure, knockdown of MEF2A reduced PD-L1 level, whereas further overexpressing PD-L1 resulted in upregulation of PD-L1 but did not influence MEF2A level (Fig. 4A). MEF2A depletion boosted the repressive role of cetuximab in cell viability, which could be overturned following PD-L1 overexpressing (Fig. 4B). MEF2A silencing was able to facilitate cetuximab-induced inhibition of cell proliferation, however, after PD-L1 upregulation, this outcome could be counteracted (Fig. 4C, D). MEF2A depletion further elevated the increased percentage of apoptotic cells elicited by cetuximab, whereas PD-L1 overexpression reversed the effect of MEF2A knockdown (Fig. 4E). Cetuximab-induced increase in cleaved caspase-3 and decreases in uncleaved PARP, cyclin D1, cyclin E1 and CDK2 were able to be further enhanced by silencing of MEF2A, whereas these outcomes were antagonized following upregulation of PD-L1 (Fig. 4F). The outcomes confirmed knockdown of MEF2A boosted cetuximab sensitivity by downregulating PD-L1.

### PD-L1 bound to SOX12 mRNA and stabilized its expression

**Fig. 5:**
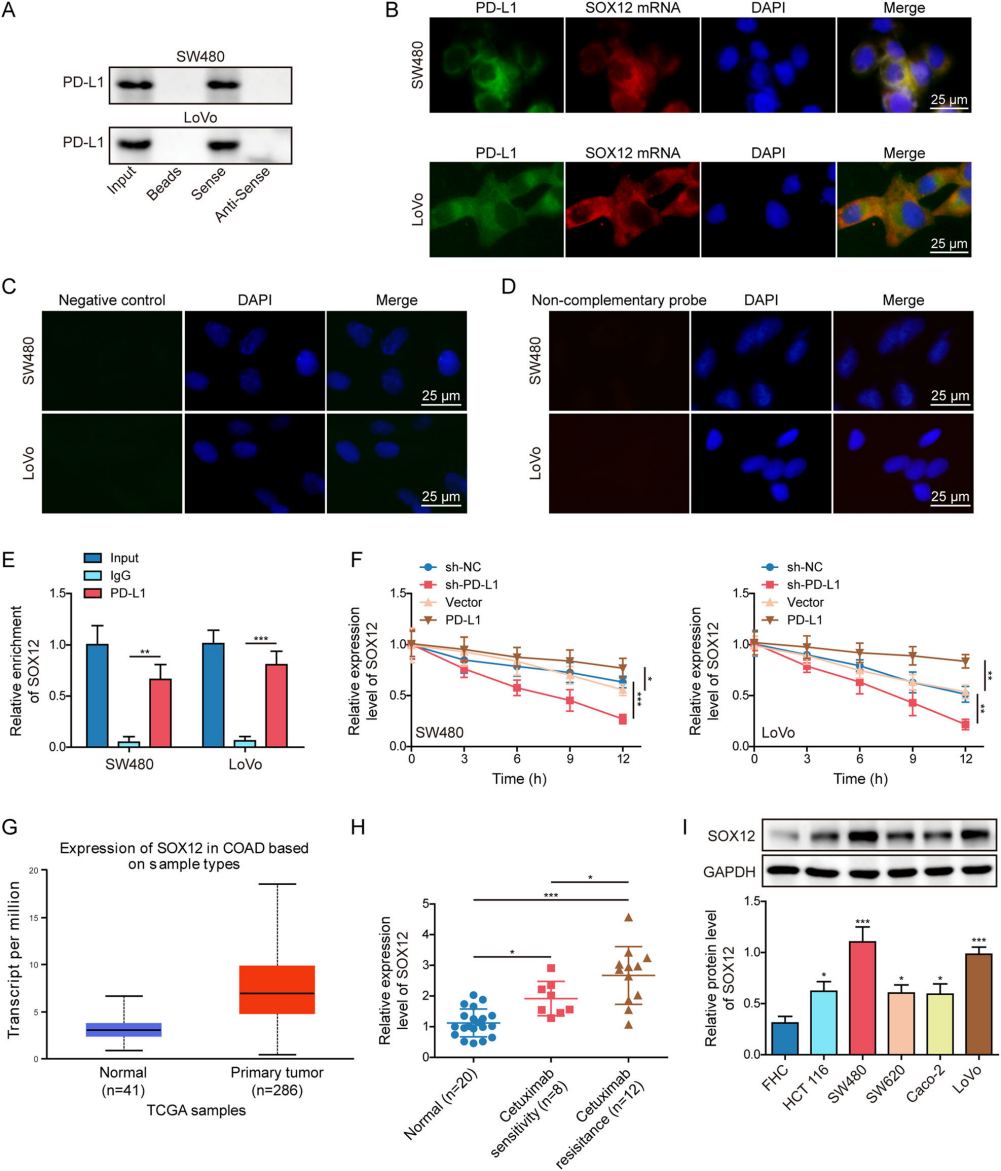
PD-L1 bound to SOX12 mRNA and stabilized its expression. **A** PD-L1 protein pulled down by biotinylated SOX12 probe in RNA pull-down assay. **B** The co-localization of SOX12 mRNA and PD-L1 was measured using FISH combined with immunofluorescence. Scale bar: 25 μm. Representative immunofluorescence (**C**) and FISH (**D**) images in the negative control experiments. Scale bar: 25 μm. **E** RIP for detecting the binding relationship between PD-L1 and SOX12 mRNA. **F** Actinomycin D was added into CRC cells transfected with sh-PD-L1 or PD-L1 overexpression vector. SOX12 mRNA expressions were tested using RT-qPCR. **G** SOX12 level in CRC was analyzed using bioinformatics analysis (UALCAN). **H** SOX12 mRNA levels in normal tissues (*n* = 20), cetuximab-sensitive (*n* = 8) and cetuximab-resistant (*n* = 12) CRC tissues were detected with RT-qPCR. **I** Western blot for determining SOX12 protein level in CRC cells. All bar chart analysis of western blot is a repeated experiment three times. *p < 0.05, **p < 0.01, and ***p < 0.001.

We further examined the underlying mechanism by which PD-L1 involved in cetuximab sensitivity. RNA pull-down results showed the biotinylated SOX12 probe could pull down PD-L1 protein in SW480 and LoVo cells (Fig. 5A). The subcellular localization of PD-L1 and SOX12 mRNA was detected, which illustrated the co-localization of PD-L1 and SOX12 mRNA in the cytoplasm (Fig. 5B). Additionally, negative control experiments were performed to ensure the reliability of the immunofluorescence and FISH results, which showed that almost no fluorescence was observed in the negative control groups of immunofluorescence (Fig. 5C) and FISH (Fig. 5D). Furthermore, RIP assay confirmed the interaction between PD-L1 and SOX12 mRNA (Fig. 5E). These results indicated that PD-L1 could bind to SOX12 mRNA. Overexpression and knockdown of PD-L1 respectively inhibited and promoted the degradation of SOX12 mRNA in the presence of actinomycin D, compared to corresponding control group (Fig. 5F). Bioinformatics analysis (UALCAN) showed higher SOX12 expression in CRC tissues than that of normal tissues (Fig. 5G). Furthermore, SOX12 mRNA level was highly expressed in cetuximab-sensitive CRC tissues, besides, it was higher in resistant CRC specimens (Fig. 5H). In addition, upregulation of SOX12 protein was also observed in CRC cell lines (Fig. 5I). These results validated PD-L1 directly bound to SOX12 mRNA and stabilized its expression in CRC.

### PD-L1 knockdown enhanced cetuximab sensitivity by repressing the stability of SOX12 mRNA

**Fig. 6:**
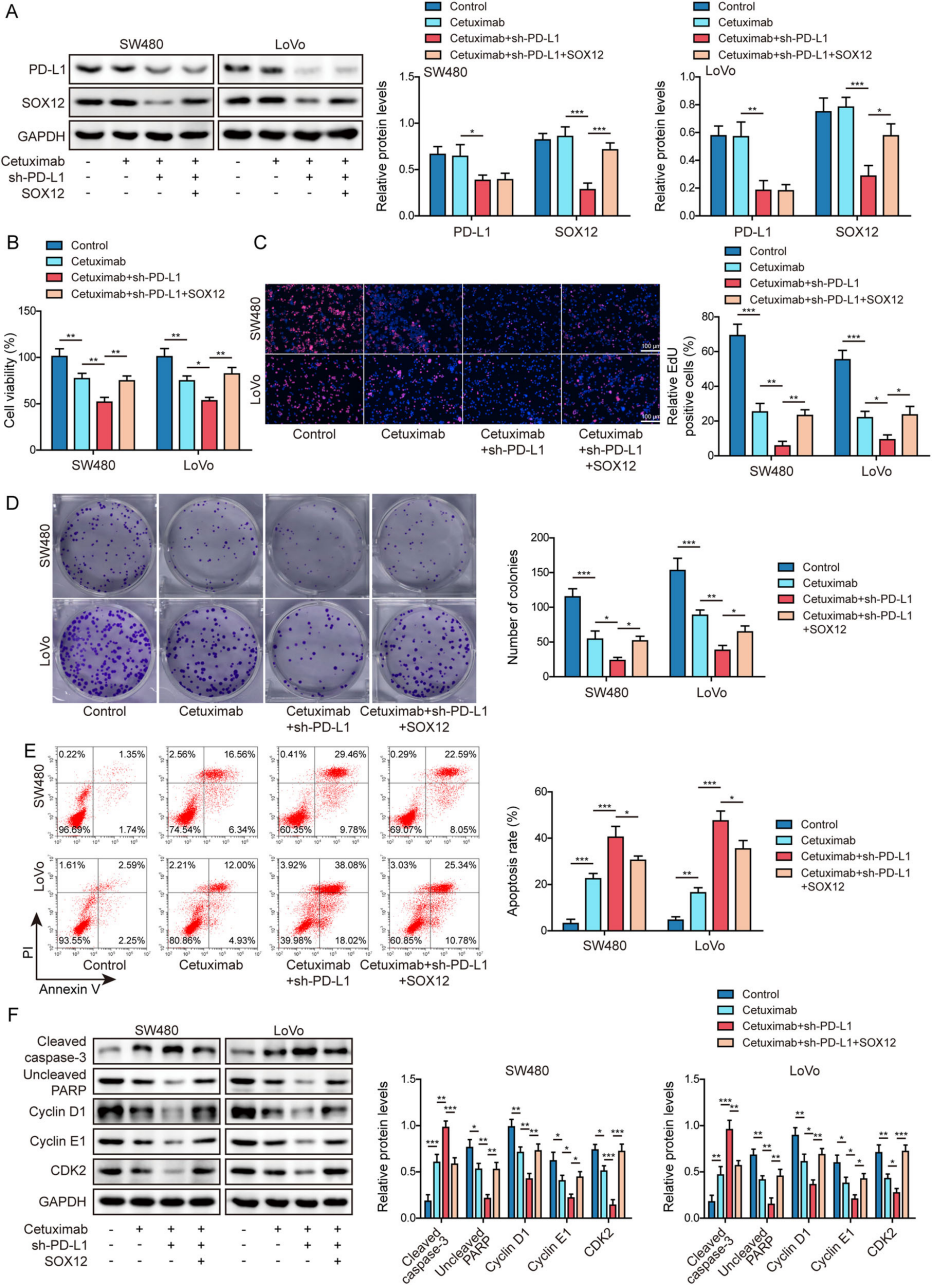
PD-L1 knockdown strengthened cetuximab sensitivity by inhibiting the stability of SOX12 mRNA. CRC cells transfected with sh-PD-L1 or SOX12 overexpression vector were exposed to cetuximab. **A** Western blot for testing PD-L1 and SOX12 protein expressions. **B** CCK-8 assay for cell viability measurement. EdU staining (**C**) and clonal formation assay (**D**) for determining cell proliferation. Scale bar: 100 μm. **E** Apoptosis analyzed by flow cytometry. **F** Western blot for determining the expressions of cleaved caspase-3, uncleaved PARP, cyclin D1, cyclin E1 and CDK2. All bar chart analysis of western blot is a repeated experiment three times. *p < 0.05, **p < 0.01, and ***p < 0.001.

Next, we examined whether PD-L1/SOX12 axis played a role in cetuximab sensitivity. CRC cells transfected with sh-PD-L1 and SOX12 overexpression vector, were exposed to cetuximab. Cetuximab treatment did not affect PD-L1 and SOX12 expression levels in comparison with control group, PD-L1 knockdown resulted in a decrease in SOX12, however, PD-L1 expression was not altered by SOX12 overexpression (Fig. 6A). When depleting PD-L1 in CRC cells, the suppressed cell viability caused by cetuximab was facilitated, notably, SOX12 upregulation was able to antagonize this effect (Fig. 6B). Silencing of PD-L1 efficiently boosted the anti-tumor effect of cetuximab on cell proliferation (Fig. 6C, D) and apoptosis (Fig. 6E), additionally, these effects mediated by PD-L1 knockdown could be counteracted by SOX12 upregulation (Fig. 6C–E). PD-L1 knockdown further promoted cetuximab-induced alterations in cell cycle- and apoptosis-associated proteins, however, overexpressing SOX12 overturned the effects of PD-L1 depletion (Fig. 6F). These discoveries demonstrated that depleting PD-L1 strengthened the cetuximab sensitivity of CRC cells via reduction of SOX12.

### RBM15/IGF2BP1-mediated m6A modification led to upregulation of MEF2A in cetuximab-resistant CRC tissues

**Fig. 7:**
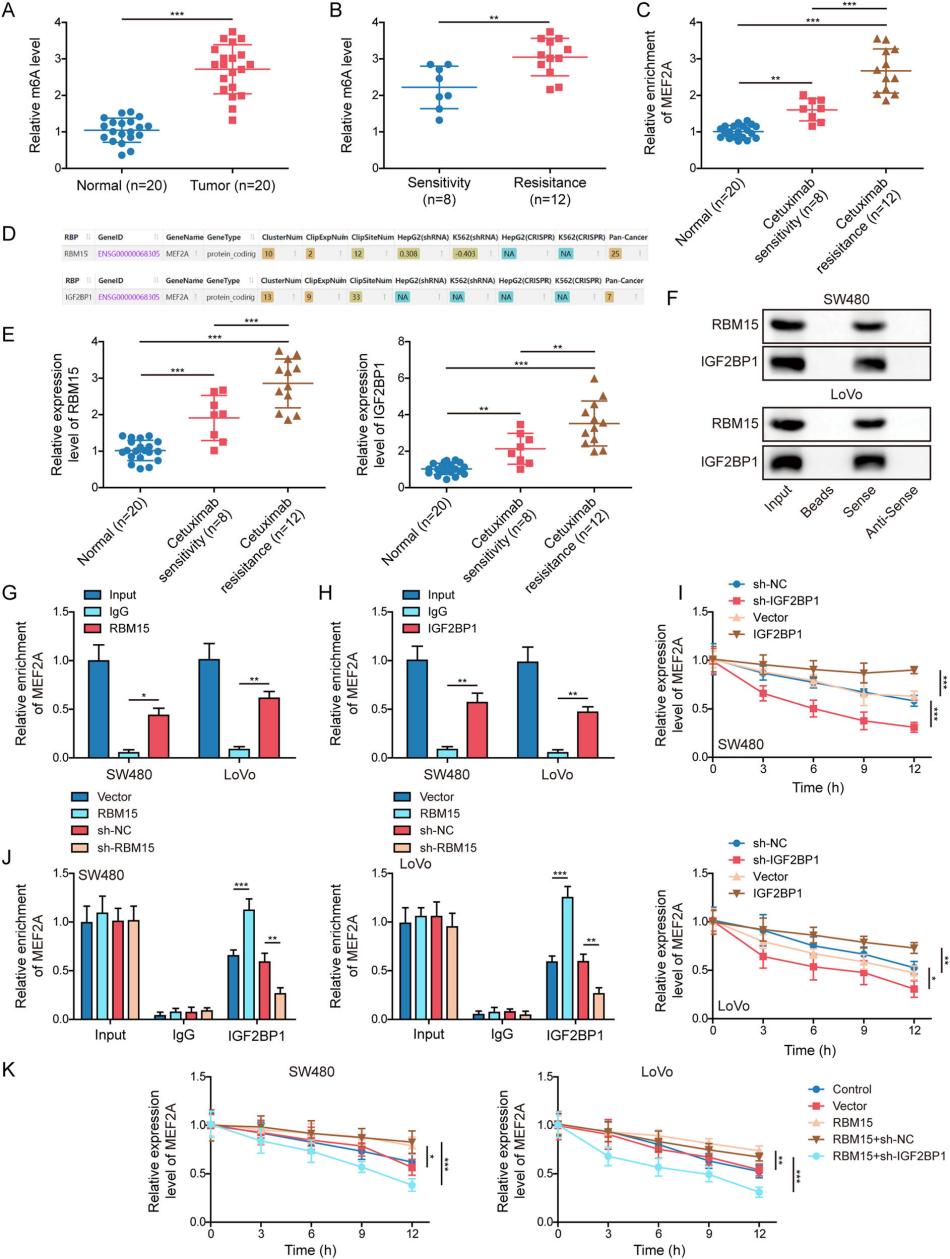
RBM15/IGF2BP1-mediated m6A modification led to upregulation of MEF2A in cetuximab-resistant CRC tissues. **A** Total m6A levels in adjacent normal tissues and cancerous tissues (*n* = 20) were tested by commercial kit. **B** Total m6A levels in cetuximab-sensitive (*n* = 8) and resistant (*n* = 12) CRC tissues. **C** MeRIP for measuring m6A modification level of MEF2A mRNA in normal tissues (*n* = 20), cetuximab-sensitive (*n* = 8) and resistant (*n* = 12) CRC tissues. **D** Bioinformatics prediction (ENCORI) showed the recognition of MEF2A mRNA by both “writer” RBM15 and “reader” IGF2BP1. **E** RT-qPCR for testing the mRNA expressions of RBM15 and IGF2BP1 in normal tissues (*n* = 20), cetuximab-sensitive (*n* = 8) and resistant (*n* = 12) CRC tissues. The combination of RBM15/IGF2BP1 with MEF2A mRNA was validated using RNA pull-down (**F**) and RIP (**G**, **H**) in CRC cells. **I** Actinomycin D was added into CRC cells transfected with sh-IGF2BP1 or IGF2BP1 overexpression vector. MEF2A mRNA level was determined by RT-qPCR. **J** CRC cells were transfected with sh-RBM15 or RBM15 overexpression vector. RIP was used to verify IGF2BP1 binding to MEF2A mRNA. **K** RBM15 overexpression vector or sh-IGF2BP1 was co-transfected into CRC cells, before actinomycin D treatment. MEF2A mRNA expression was tested by RT-qPCR. *p < 0.05, **p < 0.01, and ***p < 0.001.

The mechanism underlying the abnormal MEF2A upregulation still remains undetermined. We discovered that total m6A level was elevated in CRC tissues (Fig. 7A). Additionally, cetuximab-resistant CRC tissues presented higher m6A level than that of sensitive CRC tissues (Fig. 7B). Similar alterations were observed in the m6A modification level of MEF2A in these tissues, revealed by the data from MeRIP (Fig. 7C). Bioinformatics (ENCORI) forecasted that MEF2A mRNA could be recognized by both “writer” RBM15 and “reader” IGF2BP1 (Fig. 7D). Notably, the mRNA expressions of RBM15 and IGF2BP1 were found to be increased in cetuximab-sensitive CRC tissues, besides, they were further increased in resistant CRC tissues (Fig. 7E). RNA pull-down assay uncovered that RBM15 and IGF2BP1 were pulled down when using MEF2A mRNA probe (Fig. 7F). Moreover, RIP assay revealed that both RBM15 (Fig. 7G) and IGF2BP1 (Fig. 7H) antibodies were able to enrich MEF2A mRNA in the precipitated complexes, displaying the intermolecular interaction between RBM15/IGF2BP1 and MEF2A mRNA. In the presence of actinomycin D, overexpression and knockdown of IGF2BP1, respectively, inhibited and promoted the degradation of MEF2A mRNA, compared with corresponding control group (Fig. 7I). RIP results exhibited that RBM15 upregulation enhanced, while RBM15 depletion repressed the binding of IGF2BP1 to MEF2A mRNA (Fig. 7J). After treatment with actinomycin D, RBM15 overexpression suppressed MEF2A mRNA degradation, however, this effect could be neutralized by IGF2BP1 knockdown (Fig. 7K). The results revealed that RBM15/IGF2BP1-mediated m6A modification of MEF2A contributed to MEF2A upregulation in cetuximab-resistant CRC tissues.

### MEF2A silencing promoted cetuximab chemosensitivity in CRC cells in vivo

**Fig. 8:**
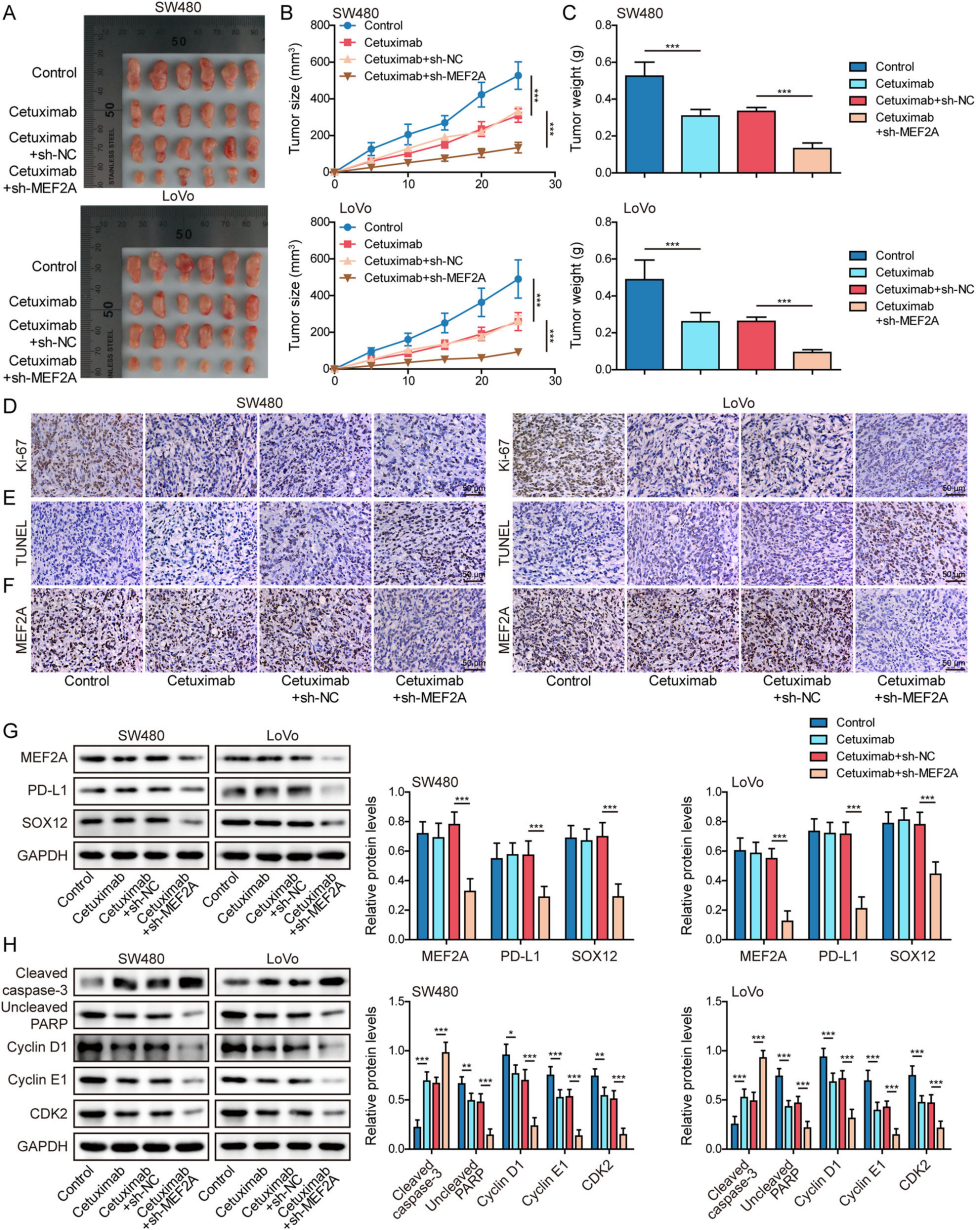
MEF2A knockdown promoted cetuximab sensitivity in CRC xenograft nude mice. CRC cells (SW480 or LoVo) with stable MEF2A knockdown were subcutaneously injected into nude mice, before cetuximab treatment. **A** Tumors formed in each group. **B** Tumor size. **C** Tumor weight. **D** Immunohistochemistry for observing Ki-67 expression in tumor tissues. Scale bar: 50 μm. **E** TUNEL staining for assessing apoptosis in tumor tissues. Scale bar: 50 μm. **F** Immunohistochemistry for measuring MEF2A expression in tumor tissues. Scale bar: 50 μm. **G** MEF2A, PD-L1, and SOX12 protein levels in tumor tissues were determined using western blot. **H** The protein expressions of cleaved caspase-3, uncleaved PARP, cyclin D1, cyclin E1, and CDK2 in tumor tissues were tested by western blot. All bar chart analysis of western blot is a repeated experiment three times. *p < 0.05, **p < 0.01, and ***p < 0.001.

By establishing subcutaneous xenograft model, the in vivo function of MEF2A on the sensitivity of cetuximab was further explored. The results demonstrated that cetuximab inhibited tumor growth in nude mice, as indicated by the decreases in tumor size and weight, meanwhile, silencing of MEF2A enhanced the efficacy of cetuximab (Fig. 8A–C). Cetuximab-treated mice presented a decrease in the expression of Ki-67, while MEF2A depletion further downregulated Ki-67 level in tumors (Fig. 8D). TUNEL staining revealed that apoptotic cells in xenograft tumors were obviously increased by the treatment of cetuximab, which were further elevated following silencing of MEF2A (Fig. 8E). Cetuximab did not affect the expression of MEF2A, PD-L1 and SOX12, whereas MEF2A knockdown reduced PD-L1 and SOX12 protein levels (Fig. 8F, G). Treatment with cetuximab led to increase of cleaved caspase-3 and declines of uncleaved PARP, cyclin D1, cyclin E1, and CDK2, notably, depleting MEF2A further strengthened the above effects induced by cetuximab (Fig. 8H). These data indicated that silencing of MEF2A sensitized CRC to cetuximab in vivo.

## Discussion

Chemoresistance is a major problem during chemotherapy in CRC, which remarkably impairs the efficiency of the treatment [22]. The long-term effectiveness of anti-EGFR therapy of cetuximab is limited due to the emergence of drug resistance [23]. Here, our research demonstrated that MEF2A silencing promoted the sensitivity of cetuximab to CRC in vitro and in vivo. MEF2A was implicated in cetuximab sensitivity in CRC through transcriptionally upregulating PD-L1 to stabilize SOX12 mRNA. Moreover, m6A modification of MEF2A mRNA mediated by RBM15/IGF2BP1 upregulated its expression in CRC tissues. For the first time, the present research confirmed MEF2A as a novel mediator for cetuximab sensitivity in CRC.

MEF2A is associated with chemotherapy resistance in many tumors. For example, MEF2A promotes resistance to cisplatin in gastric cancer [24]. Besides, upregulated MEF2A increases lncRNA SNHG16 expression, contributing to gemcitabine resistance in the starving bladder tumor microenvironment [25]. The present research confirmed that MEF2A was upregulated in CRC tissues. Especially, we first reported increased MEF2A expression in cetuximab-resistant CRC tissues. Similar results were displayed in the analysis of MEF2A level in CRC cell lines, consistently with the previous study [6]. In vivo and in vitro functional assays revealed that silencing MEF2A substantially enhanced cetuximab sensitivity to CRC cells, thus promoting the anti-tumor effect of cetuximab on CRC. This research is the first to describe MEF2A role in cetuximab sensitivity to CRC, as far as we known.

Although acting as a key factor in immune escape, PD-L1 is also involved in mediating chemotherapy resistance. PD-L1 mediates the resistance of tumor necrosis factor-related apoptosis-inducing ligand (TRAIL) in gastric cancer cells [26]. Stabilizing PD-L1 protein reduces the cisplatin sensitivity of ovarian cancer cells [27]. PD-L1 knockdown restrains cisplatin-induced chemoresistance in head and neck squamous cell carcinoma [28]. Disinhibiting PD-L1 promotes prednisone resistance of diffuse large B-cell lymphoma [29]. Cetuximab resistance can be induced by multidrug resistance cells-derived exosomes at least partly via increasing PD‑L1 expression in CRC [21]. Cetuximab resistance to CRC cells is promoted by lncRNA HCG18 via upregulating PD-L1 [15]. Based on bioinformatics analysis, we identified the MEF2A binding motif and putative binding sites of MEF2A in PD-L1 promoter region. Using luciferase reporter assay, CHIP, and EMSA, our study firstly validated MEF2A bound to PD-L1 promoter to upregulate its transcription. Notably, CRC cell lines with PD-L1 knockout exhibited an augment in cetuximab chemosensitivity. Furthermore, we verified that decreased MEF2A enhanced the sensitivity of CRC cells to cetuximab, whereas this effect was blocked by PD-L1 overexpression. This study provides evidence that PD-L1 acts as the downstream target as well as the mediator of MEF2A in the resistance of CRC cells to cetuximab.

PD-L1 acts as an RNA-binding protein that regulates the downstream mRNA stability [16, 17]. We are the first to demonstrate that PD-L1 binds to SOX12 mRNA to promote its stability, by using RNA pull-down, RIP, and FISH coupled with immunofluorescence staining. CRC cell proliferation, invasion, and migration can be facilitated via upregulating SOX12 [30, 31]. Importantly, SOX12^+^ hepatocellular carcinoma cells are more chemoresistant to cisplatin [20]. Our study further explored the involvement of PD-L1 in cetuximab sensitivity was exerted by regulating SOX12 mRNA stability. SOX12 levels in CRC cells and tissues, especially in cetuximab-resistant CRC tissues, were obviously upregulated. In the meantime, PD-L1 knockdown led to augmented chemosensitivity to CRC cells via downregulation of SOX12. Collectively, our study verified that MEF2A contributed to CRC resistance to cetuximab through regulating PD-L1/SOX12 mRNA axis.

We further explored the mechanism of MEF2A upregulation in CRC tissues. Here, our study firstly discovered high m6A modification level of MEF2A mRNA in cetuximab-resistant CRC tissues. By using bioinformatics prediction, we identified that MEF2A mRNA could be recognized by both m6A “writer” RBM15 and “reader” IGF2BP1. RBM15 and IGF2BP1 were found to be increased in CRC tissues, especially in cetuximab-resistant CRC tissues. For the first time, we validated that RBM15/IGF2BP1-mediated m6A modification of MEF2A contributed to the upregulation of MEF2A in cetuximab-resistant CRC tissues. RBM15 modulates m6A modification of YES proto-oncogene 1 (YES1) mRNA in an IGF2BP1-dependent manner, promoting hepatocellular carcinoma progression [32], indicating RBM15/IGF2BP1 regulates the m6A modification of other genes in other diseases. Furthermore, a study has proved that declined MEF2A expression can be induced by deficiency of wilms tumor 1-associated protein (WTAP), a m6A writer, in the heart [33], suggesting that MEF2A is modified by other m6A modification writer. Moreover, IGF2BP1 stabilizes other mRNAs to regulate chemotherapy resistance. For example, IGF2BP1-stabilized estrogen-related receptor alpha (ERRα) mRNA is involved in metabolic reprogramming of chemoresistant osteosarcoma cells [34]. IGF2BP1 arginine methylation mediated by protein arginine methyltransferase 3 (PRMT3) promotes oxaliplatin resistance through stabilizing the mRNA of HEG1 in liver cancer [35]. Also, a previous study has verified that RBM15 regulates Ras association domain family 8 (RASSF8) stability via m6A modification to facilitate lung adenocarcinoma progression [36], which demonstrates RBM15 mediates the m6A modification of other mRNAs.

To sum up, our data reveal that upregulated MEF2A is responsible for the inhibition of cetuximab sensitivity in CRC. Mechanistically, MEF2A induced by RBM15/IGF2BP1-mediated m6A modification can transcriptionally upregulate PD-L1 to stabilize SOX12 mRNA, thereby causing suppression of cetuximab sensitivity in CRC. These findings suggest that MEF2A may represent a promising therapeutic target for ameliorating the effectiveness of cetuximab therapy in CRC. Given that PD-L1 is associated with immune escape, whether this functional axis can affect immune escape in CRC needs further investigation.

## Materials and methods

### Clinical sample collection

Para-cancerous normal tissues and cancer samples were collected from 20 CRC patients who underwent cetuximab chemotherapy and received surgery or biopsy in Soochow University (Suzhou, Jiangsu, China). The present research was approved by the Ethics Committee of Soochow University, and informed consent was obtained from all individuals. All methods in clinical research were performed in accordance with the relevant guidelines of the Ethics Committee of Soochow University. The clinical pathological traits of these CRC patients were exhibited in Supplementary Table 1. Objective response was evaluated using the modified Response Evaluation Criteria in Solid Tumors (RECIST) criteria. Participants were followed up every 3–6 months after cetuximab therapy. Patients with partial or complete response, stable disease over 6 months were classified as cetuximab-sensitive group [37].

### Cell culture

A normal colonic epithelial cell line (FHC) and five human CRC cell lines (HCT 116, SW480, SW620, Caco-2, LoVo) were bought from the American Type Culture Collection (Manassas, VA, USA). Besides, PD-L1 knockout (KO) CRC cell lines (SW480-KO and LoVo-KO) were generated and purchased from Haixing Biotechnology Co., Ltd (Suzhou, Jiangsu, China). All cells were maintained in RPMI 1640 medium (Gibco, Grand Island, NY, USA) containing 1% penicillin/streptomycin (Gibco) and 10% fetal bovine serum (FBS, Gibco) at 37 °C in a 5% CO_2_ incubator. Cells were certificated by short tandem repeat (STR) profiling, and confirmed to be free of mycoplasma contamination.

### Cell transfection and cetuximab treatment

pcDNA3.1 vector, MEF2A, PD-L1, SOX12, IGF2BP1, and RBM15 overexpression vectors, short hairpin RNAs (shRNAs) targeting MEF2A, PD-L1, IGF2BP1, and RBM15 (sh-MEF2A, sh-PD-L1, sh-IGF2BP1, and sh-RBM15), and the negative control (sh-NC) were all synthesized from RiboBio (Guangzhou, China). Cells were planted in a 12-well plate (5 × 10^4^ cells per well) at 37 °C overnight. By using Lipofectamine 3000 reagent (Invitrogen, Carlsbad, CA, USA), the above plasmids were transfected into CRC cells, and cells were collected for subsequent analyses following 48 h. Specific sequences of shRNAs were presented in Supplementary Materials. To determine cetuximab sensitivity, cells were treated with cetuximab (2.5, 5, 10, 20, 40 or 80 μg/mL) (Merck, Darmstadt, Germany) for 48 h.

### CCK-8 assay

Cell viability was detected by using a CCK-8 Kit (Dojindo, Tokyo, Japan). In short, cells were planted in a 96-well plate, followed by cetuximab treatment for 48 h. Next, each well was added with CCK-8 reagent, then incubated at 37 °C for 4 h. A microplate reader (Thermo Fisher Scientific, Waltham, MA, USA) was used to measure the optical density at 450 nm. Dose curves were plotted to determine value of half-maximal inhibitory concentration (IC50).

### EdU staining

According to protocol of EdU Kit (Beyotime, Shanghai, China), cells were planted into a 24-well plate (1 × 10^4^ cells/well) and incubated for 12 h. After indicated treatments, each well was added with EdU reagent and incubated for 2 h at 37 °C. After fixing in paraformaldehyde (4%) for 15 min at 4 °C, cells were permeabilized for 10 min at room temperature in Triton X-100 (0.3%). Next, they were reacted for 0.5 h with Apollo reaction mixture, followed by nuclei staining for 10 min at room temperature by Hoechst 33342 (Beyotime). An inverted fluorescence microscope (Olympus, Tokyo, Japan) was used to calculate the proportion of EdU-positive cells. All operations were carried out by experimenters blinded to the experimental design.

### Colony formation assay

Cell proliferation was also detected by colony formation assay. In brief, cells were seeded in 6-well plates (400 cells/well) and cultured for 14 days. After washing with PBS, cells were fixed in methanol for 15–20 min at room temperature, then stained by crystal violet (0.5%) for 15 min at room temperature. Subsequently, colonies were counted with an optical microscope (Olympus). All operations were carried out by experimenters blinded to the experimental design.

### Flow cytometry

Cells (1 × 10^6^ cells/mL) were resuspended, then cell suspension (300 μL) was added with 5 μL Annexin V and 5 μL Propidium Iodide (PI) solution (Keygen Biotechnology, Nanjing, China), followed by incubation in the dark for 15 min at room temperature. A FACSort Flow Cytometer (BD, SanJose, CA, USA) was used to quantify the apoptotic cells.

### mRNA stability detection

Transfected cells were planted in a 6-well plate, then added with 5 µg/mL of actinomycin D (MedChemExpress, Monmouth Junction, NJ, USA) for blocking de novo transcription. After 0, 3, 6, 9, or 12 h, cells were collected to extract total RNA. Next, MEF2A and SOX12 mRNA levels were measured using RT-qPCR.

### Luciferase reporter assay

Wide-type (WT) or mutant (MUT) sequences of putative MEF2A binding sites in PD-L1 promotor region were inserted into pGL3.0 luciferase reporter vector (GenePharma, Shanghai, China). Cells were planted into 24-well plates (1 × 10^5^/well) for 12 h-culture. Next, cells were co-transfected with mutant or wide-type luciferase plasmids and MEF2A-overexpressing vector or negative control using Lipofectamine 3000 transfection reagent (Invitrogen). Following transfection for 48 h, Luciferase Reporter Assay Kit (RG005, Beyotime) was used to measure the luciferase activity.

### Chromatin immunoprecipitation (CHIP)

An EZ-Magna ChIP Kit (17-10086, Millipore, Bedford, MA, USA) was used to analyze the binding of MEF2A on the PD-L1 promoter. In brief, 1 × 10^7^ cells were cross-linked in formaldehyde (1%) for 10 min at room temperature. Lysis buffer was used to extract the chromatin, then DNA was sheared into 200–1000 bp by sonication for 10 min. Samples were immersed overnight at 4 °C with anti-MEF2A (A303-531A, Invitrogen, 1:50) or anti-IgG (ab313801, Abcam, 1:50). Protein/DNA complexes were eluted, and the cross-link of protein/DNA was reversed with 0.5 mg/mL proteinase K at 65 °C for 2 h. After being purified, the precipitated DNA fragments were quantified using PCR analysis.

### Electrophoretic mobility shift assay (EMSA)

The EMSA Kit (20148, Thermo Fisher Scientific) was used to validate the interaction between MEF2A and PD-L1 promoter region. Briefly, recombinant MEF2A protein was incubated with the PD-L1 probe (RiboBio) in binding buffer for 30 min at room temperature. Next, protein-DNA complexes were separated by electrophoresis, followed by transfer to nylon membranes. Afterwards, samples were measured with streptavidin-horseradish peroxidase and chemiluminescent substrate.

### RNA immunoprecipitation (RIP)

An EZ-Magna RIP Kit (17-701, Millipore) was used to carry out RIP assay. In brief, cell lysate was incubated overnight at 4 °C in magnetic beads conjugated with anti-PD-L1 (PDL1-101AP, Invitrogen, 1:50), anti-RBM15 (PA5-22067, Invitrogen, 1:200), anti-IGF2BP1 (PA5-89310, Invitrogen, 1:100), or anti-IgG (ab313801, Abcam, 1:50) antibody. The RNA was purified with proteinase K for 20 min at 55 °C, then RT-qPCR was performed for detecting the target RNA.

### RNA pull-down

Biotinylated probes against SOX12 or MEF2A were obtained from GenePharma. Pierce Magnetic RNA-Protein Pull-Down Kit (20164, Thermo Fisher Scientific) was used to perform RNA pull-down assay. In brief, lysis buffer was used to lyse CRC cells. Next, samples were immersed in the biotinylated probes or anti-sense probes for 2 h at room temperature, then streptavidin beads were added. Western blot analysis was performed to detect the bound proteins.

### Fluorescence in situ hybridization (FISH) and immunofluorescence

SOX12 probe labeled with Cy5 was constructed by RiboBio, and a non-complementary probe was used as negative control. Cells were fixed in 4% paraformaldehyde for 20 min at 4 °C and then permeabilized for 15 min at room temperature. FISH Kit (C10910, RiboBio) was used to perform RNA FISH assay. For immunofluorescence, cells were incubated in anti-PD-L1 antibody (PA5-20343, Invitrogen, 1:100) at 4 °C overnight. Afterwards, cells were incubated in Alexa Fluor 488 secondary antibody (ab150077, Abcam, 1:200) at room temperature for 2 h. In the negative control group, PD-L1 primary antibody was not added and other steps were exactly the same. A confocal microscope (Nikon, Tokyo, Japan) was used to detect the co-localization of SOX12 mRNA and PD-L1 in CRC cells. Image J software was used for analysis of the co-localization of PD-L1 with SOX12 mRNA. All operations were carried out by experimenters blinded to the experimental design.

### m6A modification level detection

An m6A RNA methylation quantification Kit (ab185912, Abcam) was used to measure overall m6A level. In brief, overall RNA was separated from tissues with Trizol (Invitrogen). Next, RNA sample (200 ng) was incubated with 50 µL of diluted capture antibody for 1 h at room temperature. Afterwards, detection antibody and enhancer solution were added for incubation successively. Finally, samples were incubated with developer solution for 10 min at room temperature. A microplate reader was used to detect absorbance at 450 nm.

### Methylated RNA immunoprecipitation (MeRIP)

Magna MeRIP m6A Kit (17-10499, Millipore) was used to determine the m6A modification level of MEF2A. Total RNA was extracted from tissue samples with TRIzol (Invitrogen). Then beads and m6A antibody provided in the kit were pre-mixed in IP buffer for 1 h at room temperature. For immunoprecipitation, RNA was incubated at 4 °C for 4 h in antibody-bead mixture. The precipitated mRNA was analyzed using RT-qPCR.

### Animal experiments

Male BALB/c nude mice (4–6 weeks) were purchased from the SLAC Laboratory Animal Center (Shanghai, China). Animals had free access to food and water, and were kept under specific pathogen-free condition with a 12-h light-dark cycle. Animal experiments had been approved by the Animal Care and Use Committee of Soochow University, and all methods involved in animal experiments were performed in accordance with the relevant guidelines of the Animal Care and Use Committee of Soochow University.

For stable knockdown of MEF2A expression, control lentivirus (sh-NC) and sh-MEF2A lentivirus were purchased from GenePharma. SW480 and LoVo cells were infected with lentivirus for 48 h. Afterwards, cells were selected for 14 days by puromycin (2 ng/mL, Sigma-Aldrich, St. Louis, MO, USA). Mice were randomly divided into four groups, with six mice in each group. Next, SW480 and LoVo cells stably infected with control or sh-MEF2A lentivirus were subcutaneously injected into the right flank regions of mice (5 × 10^6^ cells in 100 μL PBS per mouse). The next day, 10 mg/kg of cetuximab or vehicle (normal saline) were intraperitoneally injected into mice every 2 days for 3 weeks. A vernier caliper was used to monitor the tumor growth every 5 days after implantation, and tumor volume was calculated according to the formula: length × width^2^/2. After 25 days, animals were sacrificed to harvest the tumor bulks. The tumor weight was measured, then the tumor tissues were collected for pathological and western blot detections. All operations were carried out by experimenters blinded to the experimental design.

### TUNEL staining

Tumor tissues were fixed for 24 h in formalin (10%), and embedded with paraffin, followed by cut into 5-μm sections. In line with the manufacturer’s directions, a TUNEL Kit (12156792910, Roche, Basel, Switzerland) was used to detect apoptotic cells in tumor tissues. Images were captured under a microscope (Olympus). All operations were carried out by experimenters blinded to the experimental design.

### Immunohistochemistry

Briefly, tissue slices embedded with paraffin were dewaxed and rehydrated, followed by antigen retrieval with heated citrate buffer (0.01 mol/L) and blocking by 10% goat serum. Next, they were incubated in anti-MEF2A (A303-531A, Invitrogen, 1:500) or anti-Ki-67 (ab15580, Abcam, 1:200) antibody overnight at 4 °C. Subsequently, goat anti-rabbit IgG H&L (HRP) secondary antibody (ab6721, Abcam, 1:1000) was used to incubate the sections for 1 h at room temperature. After staining by diaminobenzidine and hematoxylin, slices were observed under a microscope (Olympus). All operations were carried out by experimenters blinded to the experimental design.

### RT-qPCR

TRIzol reagent (Invitrogen) was applied to extract total RNAs from cells and tissues. Next, PrimeScript RT reagent Kit (RR047A, Takara, Tokyo, Japan) was used for reverse transcription. RT-qPCR was carried out on MyiQ Real-Time PCR Detection System (Bio-Rad, Hercules, CA, USA) with SYBR Premix Ex Taq II reagent Kit (RR820A, Takara). The relative expressions of genes were calculated with 2^−ΔΔCT^ method, with GAPDH serving as an endogenous control.

### Western blot

Total protein was extracted from cells and tissues with RIPA buffer (Beyotime). A BCA Kit (Thermo Fisher Scientific) was used to quantify the protein concentration. Total protein (30 μg) was separated with sodium dodecyl sulfate-polyacrylamide gel electrophoresis (SDS-PAGE), followed by transferring to PVDF membranes. After blocking with 5% skimmed milk for 2 h at room temperature, membranes were immersed in primary antibodies against RBM15 (PA5-22067, Invitrogen, 1:1500), IGF2BP1 (PA5-89310, Invitrogen, 1:1000), MEF2A (A303-531A, Invitrogen, 1:2000), PD-L1 (PDL1-101AP, Invitrogen, 1:1500), SOX12 (PA5-103280, Invitrogen, 1:1000), uncleaved PARP (P248, Sigma-Aldrich, 1:3000), cleaved caspase-3 (PA5-114687, Invitrogen, 1:1500), cyclin-dependent kinase 2 (CDK2, ab32147, Abcam, 1:1000), cyclin E1 (ab211342, Abcam, 1:2000), cyclin D1 (ab134175, Abcam, 1:5000), and GAPDH (ab8245, Abcam, 1:5000) at 4 °C overnight. Afterwards, membranes were incubated with secondary antibodies (ab6721/ab205719, Abcam, 1:5000) for 2 h at room temperature. The bands were visualized with an ECL Substrate Kit (Abcam), followed by quantification using Image J software.

### Statistical analysis

All experiments were performed in at least three biological replicates, and each biological replicate contained three technical replicates. Statistical analyses of all data were conducted by GraphPad Prism 8.0. Results were stated as mean ± standard deviation (SD). All the data meet the assumption of normal distribution. The comparisons were carried out using student’s t-test in two groups, or one-way analysis of variance (ANOVA) with Tukey’s post hoc test in multiple groups. *p* < 0.05 was considered as statistical significance.

## Supplementary information

Supplementary Materials

## Data Availability

The data that support the findings of this study are available from the corresponding author upon reasonable request.
