# Supplementary material for: N6-methyladenosine modification of MEF2A weakens cetuximab sensitivity in colorectal cancer via PD-L1/SOX12 axis
Source: Cell Death Discov. 2025 Jul 1;11:294. doi: 10.1038/s41420-025-02577-8 (PMC12219012; doi:10.1038/s41420-025-02577-8)

**Supplementary Table 1 Correlation between MEF2A expression and clinical pathological traits of colorectal cancer patients**

| **Characteristics of the cases** | **MEF2A low level (n=10)** | **MEF2A high level (n=10)** | ***P* value** |
| --- | --- | --- | --- |
| **Gender** |  |  | 0.3698 |
| Male | 6 | 3 |  |
| Female | 4 | 7 |  |
| **Age (years)** |  |  | 0.3498 |
| < 50 | 5 | 2 |  |
| ≥ 50 | 5 | 8 |  |
| **Tumor size (cm)** |  |  | 0.6285 |
| < 5 | 8 | 6 |  |
| ≥ 5 | 2 | 4 |  |
| **Location** |  |  | 0.5580 |
| Distal | 2 | 4 |  |
| Middle | 3 | 3 |  |
| Proximal | 5 | 3 |  |
| **Chemotherapy** |  |  | 0.0062 |
| Sensitive | 7 | 1 |  |
| Resistant | 3 | 9 |  |
| **Histologic differentiation** |  |  | 0.0679 |
| Well or moderate | 6 | 2 |  |
| Poor | 4 | 8 |  |

**Supplementary figures and legends**

**
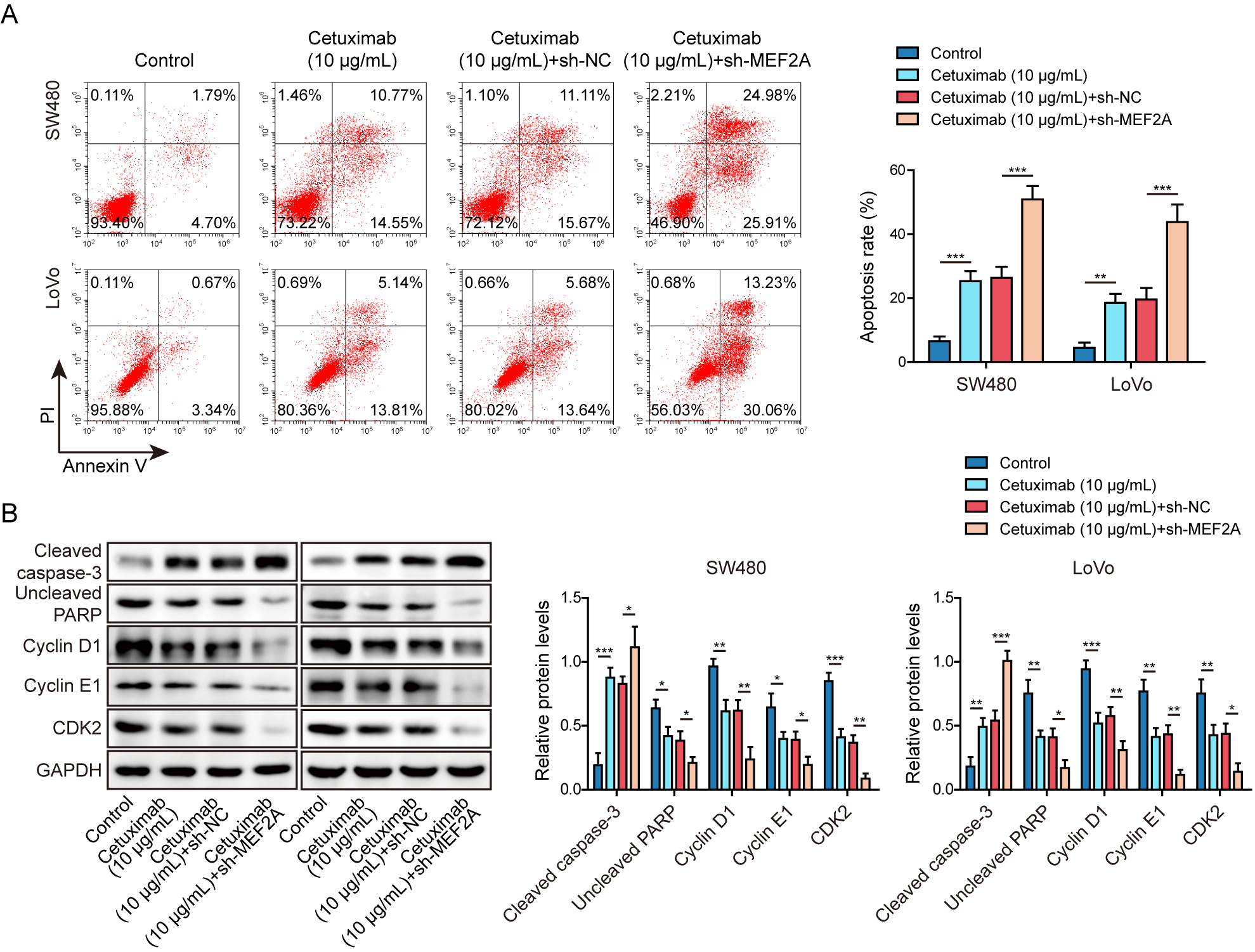
**

**Supplementary Fig. 1 Knockdown of MEF2A promoted apoptosis in CRC cells.**

CRC cells transfected with sh-MEF2A were exposed to cetuximab. (A) Flow cytometry for assessing apoptosis. (B) Apoptosis-associated proteins (cleaved caspase-3 and uncleaved PARP) and cell cycle-related proteins (CDK2, cyclin E1, and cyclin D1) were measured with western blot. All bar chart analysis of western blot is a repeated experiment three times. **p*<0.05, ***p*<0.01, and ****p*<0.001*.*

*
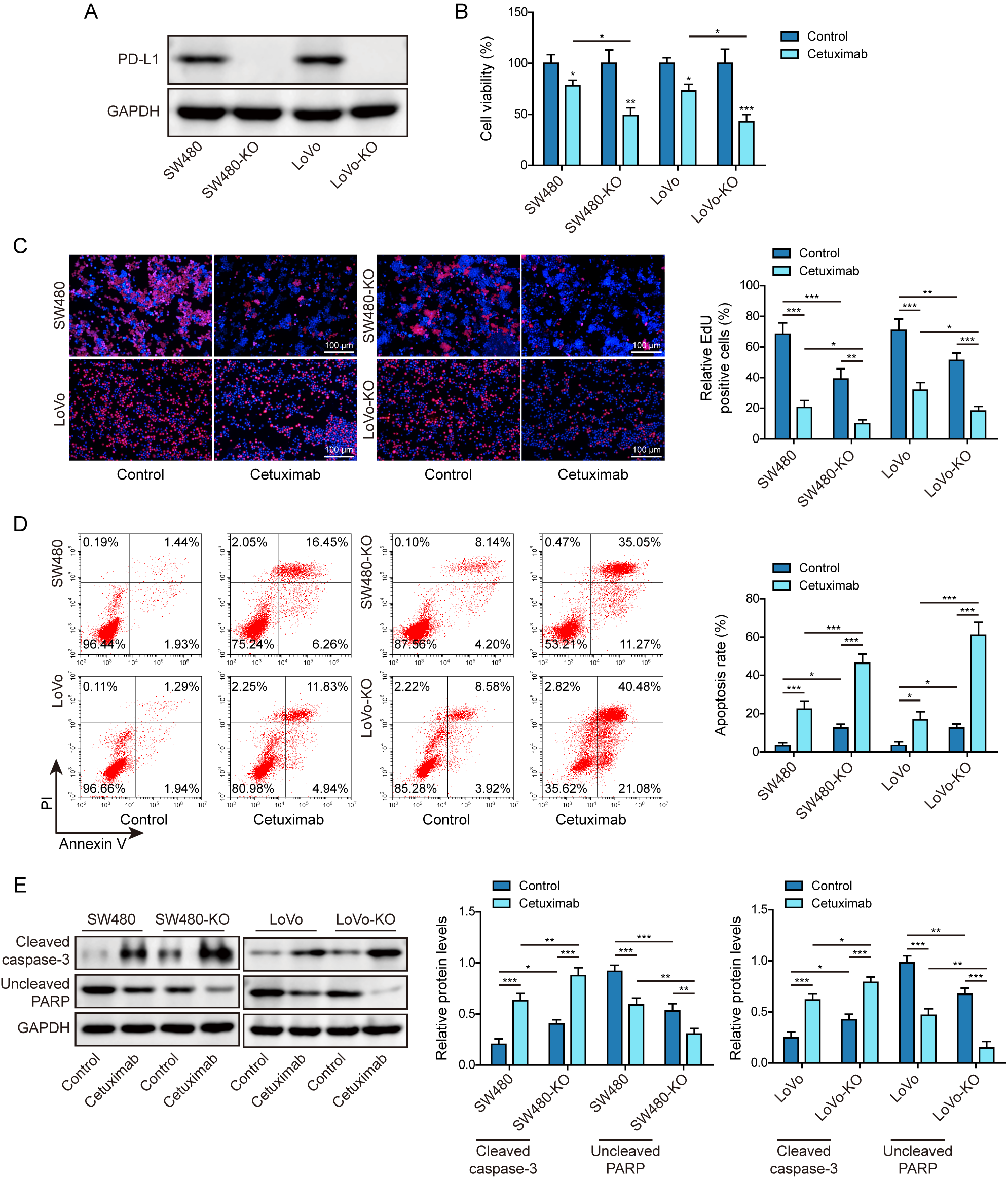
*

**Supplementary Fig. 2 Cetuximab sensitivity in PD-L1 KO cell lines.**

(A) In CRC cell lines (LoVo or SW480) and PD-L1 knockout (KO) cell lines (LoVo-KO or SW480-KO), western blot was used to measure PD-L1 protein expressions. (B-E) Cells were subjected to cetuximab or not. Cell viability determined by CCK-8 assay (B). EdU staining for detection of cell proliferation, scale bar: 100 μm. (C). Cell apoptosis evaluated with flow cytometry (D). Western blot was performed for detecting cleaved caspase-3 and uncleaved PARP protein levels (E). All bar chart analysis of western blot is a repeated experiment three times. **p*<0.05, ***p*<0.01, and ****p*<0.001*.*

**Specific sequences of shRNAs**

# **Homo sapiens myocyte enhancer factor 2A (MEF2A), transcript variant 1, mRNA**

**NCBI Reference Sequence: NM_005587.6**

atggggcggaagaaaatacaaatcacacgcataatggatgaaaggaaccgacaggtcacttttacaaagagaaagtttggattaatgaagaaagcctatgaacttagtgtgctctgtgactgtgaaatagcactcatcattttcaacagctctaacaaactgtttcaatatgctagcactgatatggacaaagttcttctcaagtatacagaatataatgaacctcatgaaagcagaaccaactcggatattgttgaggctctgaacaagaaggaacacagagggtgcgacagcccagaccctgatacttcatatgtgctaactccacatacagaagaaaaatataaaaaaattaatgaggaatttgataatatgatgcggaatcataaaatcgcacctggtctgccacctcagaacttttcaatgtctgtcacagttccagtgaccagccccaatgctttgtcctacactaacccagggagttcactggtgtccccatctttggcagccagctcaacgttaacagattcaagcatgctctctccacctcaaaccacattacatagaaatgtgtctcctggagctcctcagagaccaccaagtactggcaatgcaggtgggatgttgagcactacagacctcacagtgccaaatggagctggaagcagtccagtggggaatggatttgtaaactcaagagcttctccaaatttgattggagctactggtgcaaatagcttaggcaaagtcatgcctacaaagtctccccctccaccaggtggtggtaatcttggaatgaacagtaggaaaccagatcttcgagttgtcatccccccttcaagcaagggcatgatgcctccactaaatacccagaggatcagtagttctcaagccactcaacctcttgctaccccagtcgtgtctgtgacaaccccaagcttgcctccgcaaggacttgtgtactcagcaatgccgactgcctacaacactgattattcactgaccagcgctgacctgtcagcccttcaaggcttcaactcgccaggaatgctgtcgctgggacaggtgtcggcctggcagcagcaccacctaggacaagcagccctcagctctcttgttgctggagggcagttatctcagggttccaatttatccattaataccaaccaaaacatcagcatcaagtccgaaccgatttcacctcctcgggatcgtatgaccccatcgggcttccagcagcagcagcagcagcagcagcagcagcagccgccgccaccaccgcagccccagccacaacccccgcagccccagccccgacaggaaatggggcgctcccctgtggacagtctgagcagctctagtagctcctatgatggcagtgatcgggaggatccacggggcgacttccattctccaattgtgcttggccgacccccaaacactgaggacagagaaagcccttctgtaaagcgaatgaggatggacgcgtgggtgacctaa

Target: CCAGACCCTGATACTTCATAT

| S | AATTGCCAGACCCTGATACTTCATATTCAAGAGatatgaagtatcagggtctggTTTTTT |
| --- | --- |
| A | GATCAAAAAACCAGACCCTGATACTTCATATCTCTTGAatatgaagtatcagggtctggC |

NC：TTCTCCGAACGTGTCACGT

| S | AATTGTTCTCCGAACGTGTCACGTTCAAGAGACGTGACACGTTCGGAGAATTTTTT |
| --- | --- |
| A | GATCAAAAAATTCTCCGAACGTGTCACGTCTCTTGAACGTGACACGTTCGGAGAAC |

# **Homo sapiens CD274 molecule (CD274, also called PD-L1), transcript variant 1, mRNA**

# **NCBI Reference Sequence: NM_014143.4**

atgaggatatttgctgtctttatattcatgacctactggcatttgctgaacgcatttactgtcacggttcccaaggacctatatgtggtagagtatggtagcaatatgacaattgaatgcaaattcccagtagaaaaacaattagacctggctgcactaattgtctattgggaaatggaggataagaacattattcaatttgtgcatggagaggaagacctgaaggttcagcatagtagctacagacagagggcccggctgttgaaggaccagctctccctgggaaatgctgcacttcagatcacagatgtgaaattgcaggatgcaggggtgtaccgctgcatgatcagctatggtggtgccgactacaagcgaattactgtgaaagtcaatgccccatacaacaaaatcaaccaaagaattttggttgtggatccagtcacctctgaacatgaactgacatgtcaggctgagggctaccccaaggccgaagtcatctggacaagcagtgaccatcaagtcctgagtggtaagaccaccaccaccaattccaagagagaggagaagcttttcaatgtgaccagcacactgagaatcaacacaacaactaatgagattttctactgcacttttaggagattagatcctgaggaaaaccatacagctgaattggtcatcccagaactacctctggcacatcctccaaatgaaaggactcacttggtaattctgggagccatcttattatgccttggtgtagcactgacattcatcttccgtttaagaaaagggagaatgatggatgtgaaaaaatgtggcatccaagatacaaactcaaagaagcaaagtgatacacatttggaggagacgtaa

Target: CGAATTACTGTGAAAGTCAAT

| S | AATTGCGAATTACTGTGAAAGTCAATTCAAGAGattgactttcacagtaattcgTTTTTT |
| --- | --- |
| A | GATCAAAAAACGAATTACTGTGAAAGTCAATCTCTTGAattgactttcacagtaattcgC |

NC：TTCTCCGAACGTGTCACGT

| S | AATTGTTCTCCGAACGTGTCACGTTCAAGAGACGTGACACGTTCGGAGAATTTTTT |
| --- | --- |
| A | GATCAAAAAATTCTCCGAACGTGTCACGTCTCTTGAACGTGACACGTTCGGAGAAC |

# **Homo sapiens insulin like growth factor 2 mRNA binding protein 1 (IGF2BP1), transcript variant 1, mRNA**

# **NCBI Reference Sequence: NM_006546.4**

atgaacaagctttacatcggcaacctcaacgagagcgtgacccccgcggacttggagaaagtgtttgcggagcacaagatctcctacagcggccagttcttggtcaaatccggctacgccttcgtggactgcccggacgagcactgggcgatgaaggccatcgaaactttctccgggaaagtagaattacaaggaaaacgcttagagattgaacattcggtgcccaaaaaacaaaggagccggaaaattcaaatccgaaatattccaccccagctccgatgggaagtactggacagcctgctggctcagtatggtacagtagagaactgtgagcaagtgaacaccgagagtgagacggcagtggtgaatgtcacctattccaaccgggagcagaccaggcaagccatcatgaagctgaatggccaccagttggagaaccatgccctgaaggtctcctacatccccgatgagcagatagcacagggacctgagaatgggcgccgagggggctttggctctcggggtcagccccgccagggctcacctgtggcagcgggggccccagccaagcagcagcaagtggacatcccccttcggctcctggtgcccacccagtatgtgggtgccattattggcaaggagggggccaccatccgcaacatcacaaaacagacccagtccaagatagacgtgcataggaaggagaacgcaggtgcagctgaaaaagccatcagtgtgcactccacccctgagggctgctcctccgcttgtaagatgatcttggagattatgcataaagaggctaaggacaccaaaacggctgacgaggttcccctgaagatcctggcccataataactttgtagggcgtctcattggcaaggaaggacggaacctgaagaaggtagagcaagataccgagacaaaaatcaccatctcctcgttgcaagaccttaccctttacaaccctgagaggaccatcactgtgaagggggccatcgagaattgttgcagggccgagcaggaaataatgaagaaagttcgggaggcctatgagaatgatgtggctgccatgagcctgcagtctcacctgatccctggcctgaacctggctgctgtaggtcttttcccagcttcatccagcgcagtcccgccgcctcccagcagcgttactggggctgctccctatagctcctttatgcaggctcccgagcaggagatggtgcaggtgtttatccccgcccaggcagtgggcgccatcatcggcaagaaggggcagcacatcaaacagctctcccggtttgccagcgcctccatcaagattgcaccacccgaaacacctgactccaaagttcgtatggttatcatcactggaccgccagaggcccaattcaaggctcagggaagaatctatggcaaactcaaggaggagaacttctttggtcccaaggaggaagtgaagctggagacccacatacgtgtgccagcatcagcagctggccgggtcattggcaaaggtggaaaaacggtgaacgagttgcagaatttgacggcagctgaggtggtagtaccaagagaccagacccctgatgagaacgaccaggtcatcgtgaaaatcatcggacatttctatgccagtcagatggctcaacggaagatccgagacatcctggcccaggttaagcagcagcatcagaagggacagagtaaccaggcccaggcacggaggaagtga

Target: GCAGTGGTGAATGTCACCTAT

| S | AATTGGCAGTGGTGAATGTCACCTATTCAAGAGataggtgacattcaccactgcTTTTTT |
| --- | --- |
| A | GATCAAAAAAGCAGTGGTGAATGTCACCTATCTCTTGAataggtgacattcaccactgcC |

NC：TTCTCCGAACGTGTCACGT

| S | AATTGTTCTCCGAACGTGTCACGTTCAAGAGACGTGACACGTTCGGAGAATTTTTT |
| --- | --- |
| A | GATCAAAAAATTCTCCGAACGTGTCACGTCTCTTGAACGTGACACGTTCGGAGAAC |

# **Homo sapiens RNA binding motif protein 15 (RBM15), transcript variant 1, mRNA**

# **NCBI Reference Sequence: NM_022768.5**

atgaggactgcggggcgggaccctgtgccgcggcggagtccaagatggcggcgtgcggttccgctgtgtgaaacgagcgcggggcggcgggttactcagctccgcggagacgacctccgacgacccgcaacaatgaagggaaaagagcgctcgccagtgaaggccaaacgctcccgtggtggtgaggactcgacttcccgcggtgagcggagcaagaagttagggggctctggtggcagcaatgggagcagcagcggaaagaccgatagcggcggtgggtcgcggcggagtctccacctggacaagtccagcagtcgaggtggcagccgcgagtatgataccggtgggggcagctccagtagccgcttgcatagttatagctccccgagcaccaaaaattcttcgggcgggggcgaatcgcgcagcagctcccggggtggaggcggggagtcacgttcctctggggccgcctcctcagctcccggcggcggggacggcgcggaatacaagactctgaagataagcgagttggggtcccagcttagtgacgaagcggtggaggacggcctgtttcatgagttcaaacgcttcggtgatgtaagtgtgaaaatcagtcatctgtcgggttctggcagcggggatgagcgggtagcctttgtgaacttccggcggccagaggacgcgcgggcggccaagcatgccagaggccgcctggtgctctatgaccggcctctgaagatagaagctgtgtatgtgagccggcgccgcagccgctcccctttagacaaagatacttatcctccatcagccagtgtggtcggggcctctgtaggtggtcaccggcacccccctggaggtggtggaggccagagatcactttcccctggtggcgctgctttgggatacagagactaccggctgcagcagttggctcttggccgcctgccccctccacctccgccaccattgcctcgagacctggagagagaaagagactacccgttctatgagagagtgcgccctgcatacagtcttgagccaagggtgggagctggagcaggtgctgctcctttcagagaagtggatgagatttcacccgaggatgatcagcgagctaaccggacgctcttcttgggcaacctagacatcactgtaacggagagtgatttaagaagggcgtttgatcgctttggagtcatcacagaagtagatatcaagaggccttctcgcggccagactagtacttacggctttctcaaatttgagaacttagatatgtctcaccgggccaaattagcaatgtctggcaaaattataattcggaatcctatcaaaattggttatggtaaagctacacccaccacccgcctctgggtgggaggcctgggaccttgggttcctcttgctgccctggcacgagaatttgatcgatttggcaccatacgcaccatagactaccgaaaaggtgatagttgggcatatatccagtatgaaagcctggatgcagcgcatgctgcctggacccatatgcggggcttcccacttggtggcccagatcgacgccttagagtagactttgccgacaccgaacatcgttaccagcagcagtatctgcagcctctgcccttgactcattatgagctggtgacagatgcttttggacatcgggcaccagaccctttgaggggtgctcgggataggacaccacccttactatacagagatcgtgatagggacctttatcctgactctgattgggtgccacccccacccccagtccgagaacgcagcactcggactgcagctacttctgtgcctgcttacgagccactggatagcctagatcgcaggcgggatggttggtccttggaccgggacagaggtgatcgagatctgcccagcagcagagaccagcctaggaagcgaaggctgcctgaggagagtggaggacgtcatctggataggtctcctgagagtgaccgcccacgaaaacgtcactgcgctccttctcctgaccgcagtccagaattgagcagtagccgggatcgttacaacagcgacaatgatcgatcttcccgtcttctcttggaaaggccctctccaatcagagacagacgaggtagtttggagaagagccagggtgacaagcgagaccgtaaaaactctgcatcagctgaacgagataggaagcaccggacaactgctcccactgagggaaaaagccctctgaaaaaagaagaccgctctgatgggagtgcacctagcaccagcactgcttcctccaagctgaagtccccgtcccagaaacaggatggggggacagcccctgtggcatcagcctctcccaaactctgtttggcctggcagggcatgcttctactgaagaacagcaactttccttccaacatgcatctgttgcagggtgacctccaagtggctagtagtcttcttgtggagggttcaactggaggcaaagtggcccagctcaagatcactcagcgtctccgtttggaccagcccaagttggatgaagtaactcgacgcatcaaagtagcagggcccaatggttatgccattcttttggctgtgcctggaagttctgacagccggtcctcctcttcctcagctgcatcagacactgccacttctactcagaggccacttaggaaccttgtgtcctatttaaagcaaaagcaggcagccggggtgatcagcctccctgtggggggcaacaaagacaaggaaaacaccggggtccttcatgccttcccaccttgtgagttctcccagcagttcctggattcccctgccaaggcactggccaaatctgaagaagattacctggtcatgatcattgtccgtgggtttggttttcagataggagttaggtatgagaacaagaagagagaaaacttggcgctgaccctgttatag

Target: CCCAATGGTTATGCCATTCTT

| S | AATTGCCCAATGGTTATGCCATTCTTTCAAGAGaagaatggcataaccattgggTTTTTT |
| --- | --- |
| A | GATCAAAAAACCCAATGGTTATGCCATTCTTCTCTTGAaagaatggcataaccattgggC |

NC：TTCTCCGAACGTGTCACGT

| S | AATTGTTCTCCGAACGTGTCACGTTCAAGAGACGTGACACGTTCGGAGAATTTTTT |
| --- | --- |
| A | GATCAAAAAATTCTCCGAACGTGTCACGTCTCTTGAACGTGACACGTTCGGAGAAC |

**Original western blots**


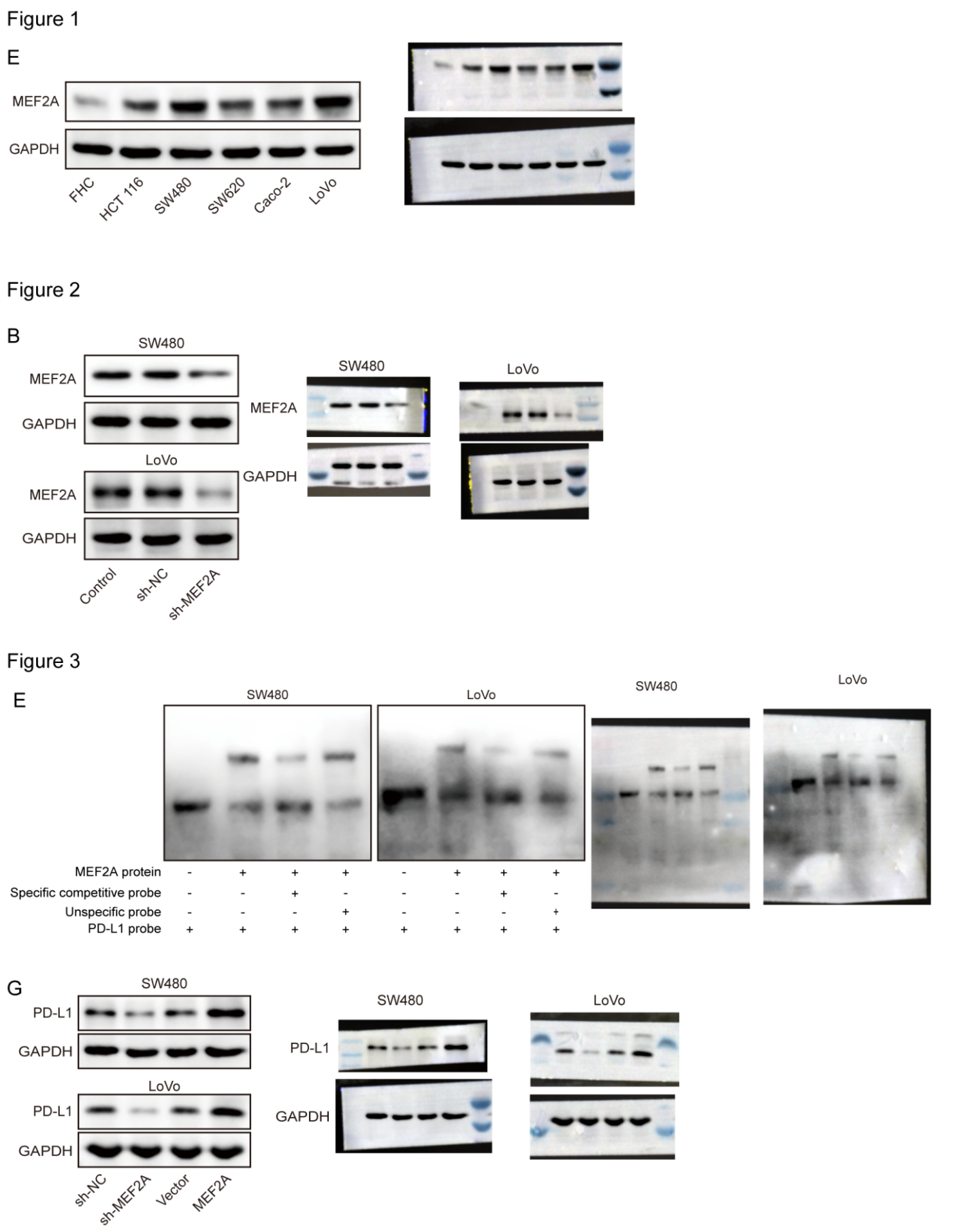


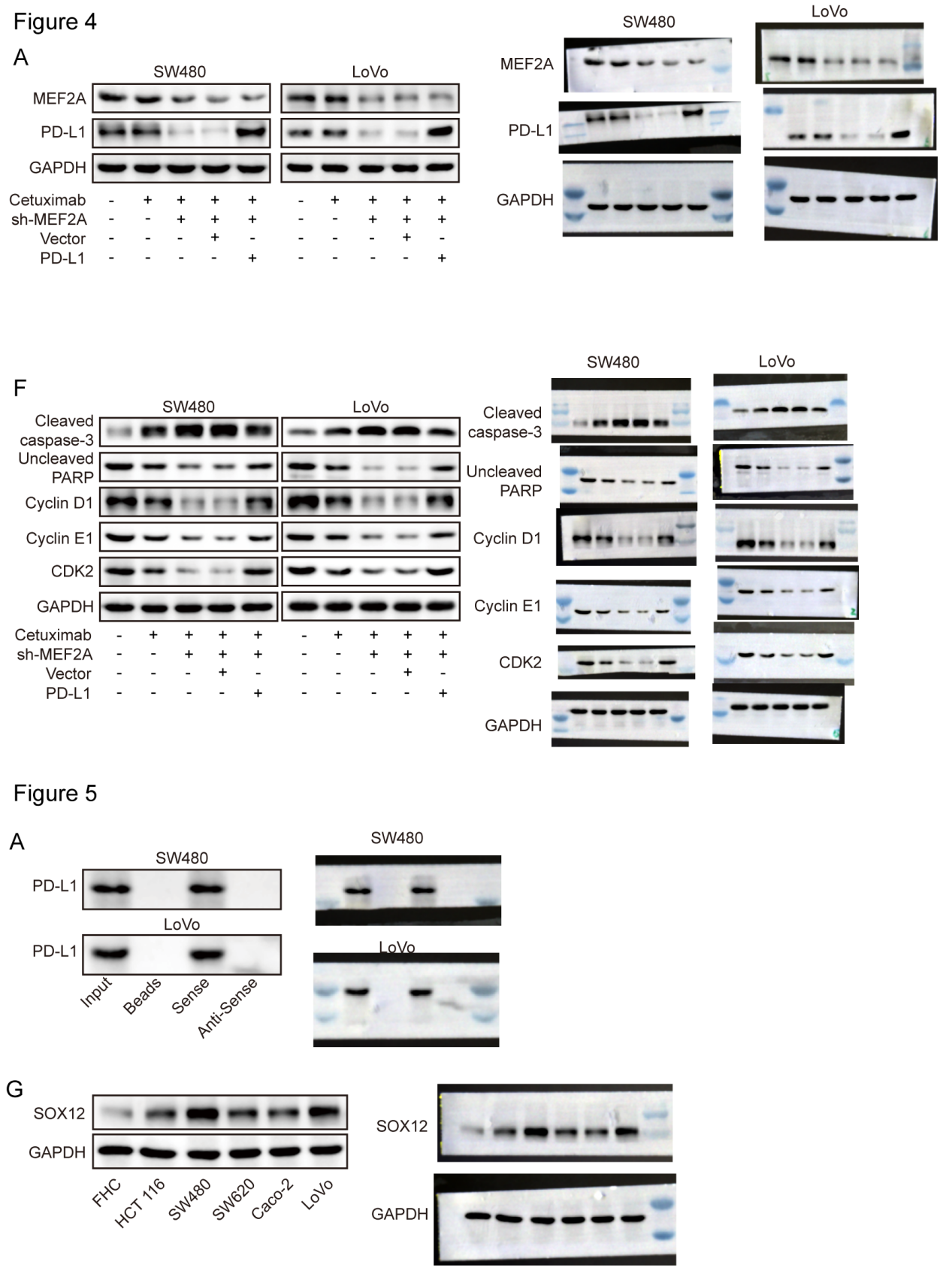


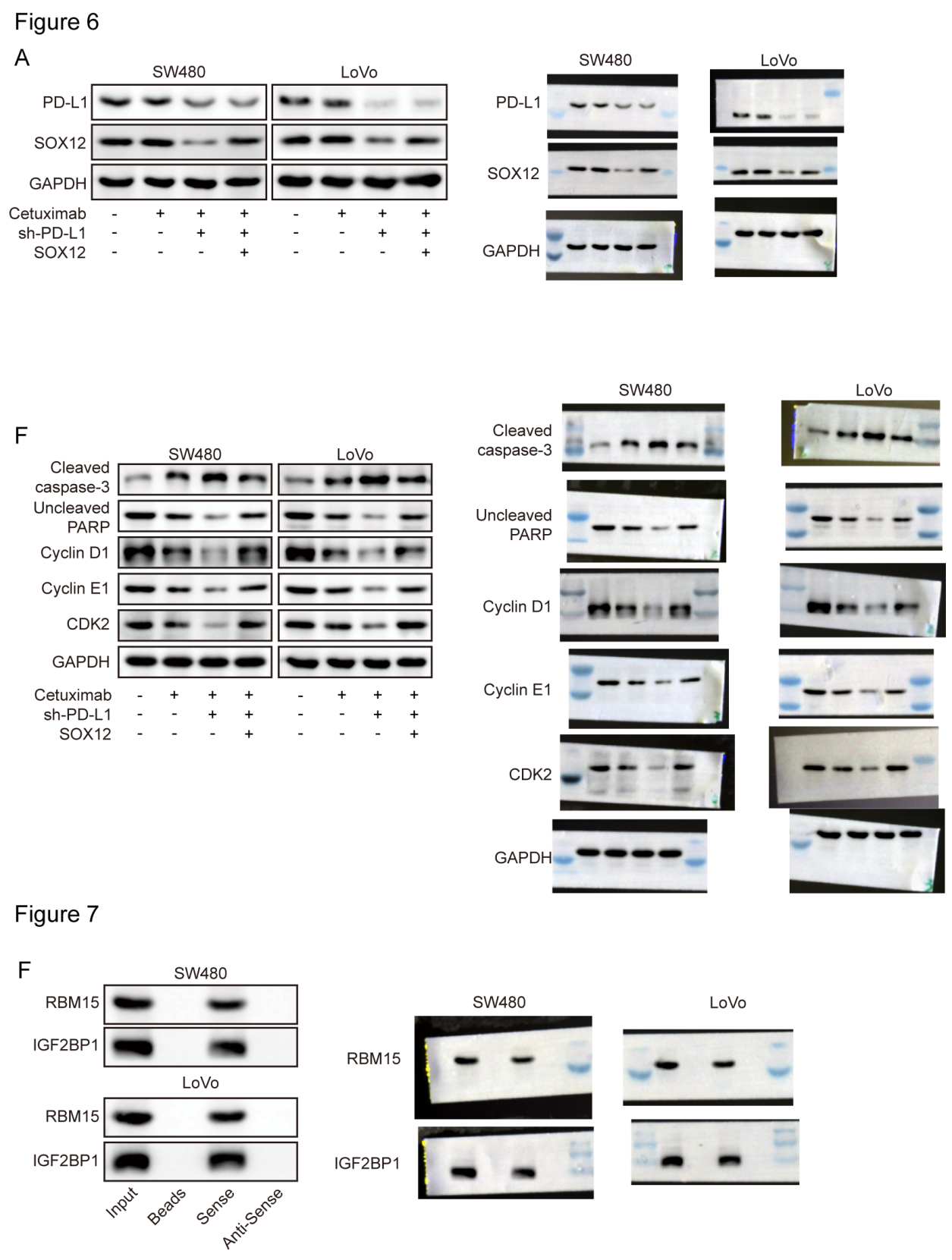


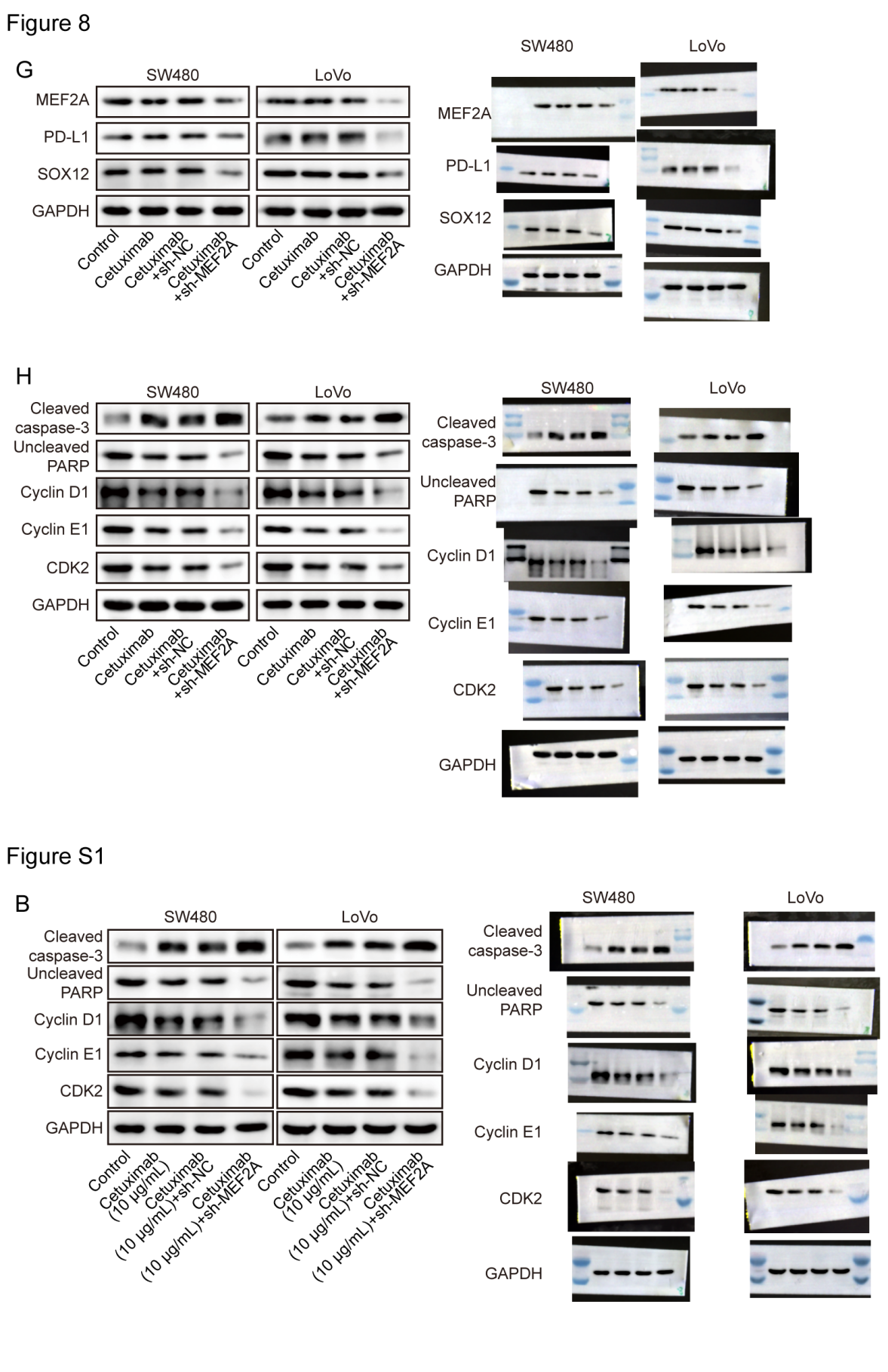


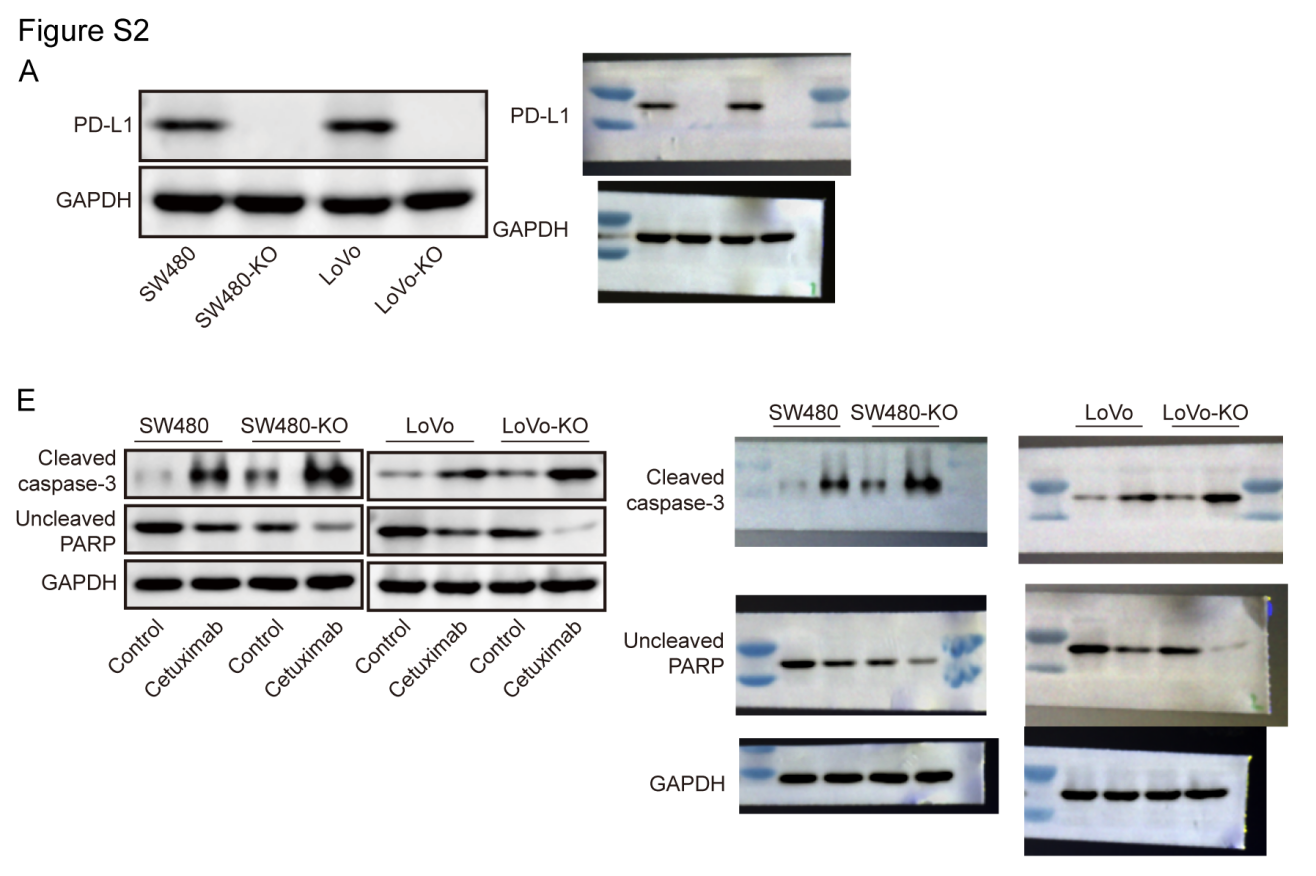

Supplement: Supplementary file 1 — Supplementary Materials [file 41420_2025_2577_MOESM1_ESM.docx]
